# Review and Assessment of Fatigue Delamination Damage of Laminated Composite Structures

**DOI:** 10.3390/ma16247677

**Published:** 2023-12-16

**Authors:** Jinghui Deng, Jie Zhou, Tangzhen Wu, Zhengliang Liu, Zhen Wu

**Affiliations:** 1 China Helicopter Research and Development Institute, Jingdezhen 333001, China; djhz5421@163.com (J.D.); wutangzhenwtz@163.com (T.W.); 2 School of Aeronautics, Northwestern Polytechnical University, Xi’an 710072, China; jie.zhou@mail.nwpu.edu.cn (J.Z.); liuzhengliang@mail.nwpu.ecu.cn (Z.L.)

**Keywords:** laminated structures, fatigue delamination, phenomenological model, Paris law, cohesive element, numerical methods

## Abstract

Fatigue delamination damage is one of the most important fatigue failure modes for laminated composite structures. However, there are still many challenging problems in the development of the theoretical framework, mathematical/physical models, and numerical simulation of fatigue delamination. What is more, it is essential to establish a systematic classification of these methods and models. This article reviews the experimental phenomena of delamination onset and propagation under fatigue loading. The authors reviewed the commonly used phenomenological models for laminated composite structures. The research methods, general modeling formulas, and development prospects of phenomenological models were presented in detail. Based on the analysis of finite element models (FEMs) for laminated composite structures, several simulation methods for fatigue delamination damage models (FDDMs) were carefully classified. Then, the whole procedure, range of applications, capability assessment, and advantages and limitations of the models, which were based on four types of theoretical frameworks, were also discussed in detail. The theoretical frameworks include the strength theory model (SM), fracture mechanics model (FM), damage mechanics model (DM), and hybrid model (HM). To the best of the authors’ knowledge, the FDDM based on the modified Paris law within the framework of hybrid fracture and damage mechanics is the most effective method so far. However, it is difficult for the traditional FDDM to solve the problem of the spatial delamination of complex structures. In addition, the balance between the cost of acquiring the model and the computational efficiency of the model is also critical. Therefore, several potential research directions, such as the extended finite element method (XFEM), isogeometric analysis (IGA), phase-field model (PFM), artificial intelligence algorithm, and higher-order deformation theory (HODT), have been presented in the conclusions. Through validation by investigators, these research directions have the ability to overcome the challenging technical issues in the fatigue delamination prediction of laminated composite structures.

## 1. Introduction

Composite materials have many advantages, such as high specific strength and flexible designability. With the development of preparation and detection technology for composite materials, different kinds of composite materials have been widely employed in civil aircraft. The composite materials that are applied in B757 and B767 account for 4%, which is 11% in B777 and even 50% in B787. Furthermore, the percentage of composite materials in A350 has amazingly reached 52%. In pursuit of lightweight materials for aircraft, there has been a tendency to use more and more advanced composite materials. Thus, the application of composite materials has become one of the important indicators to measure the performance of aircraft. Although composite materials have many advantages, there are also many problems that need to be solved in the servicing process.

Delamination is one of the most important damage modes of laminated composite structures. Delamination is very dangerous, as it is visually undetectable within laminated composite structures. Once delamination occurs, the stiffness and strength of the structure will significantly degrade, especially under compression mode. While the composite laminated structures of aircraft are subjected to complex fatigue loading conditions, fatigue delamination propagation is extremely difficult to detect and monitor. Moreover, the damage caused by fatigue delamination is irreversible and often leads to a serious decline in the performance of components and even flight accidents. Therefore, research on the initiation and propagation law [1,2], the failure mechanism [3,4], and the numerical simulation [5,6] of delamination under fatigue loads is important for the damage tolerance and durability design of composite laminated structures.

Fiber-reinforced polymer (FRP) composites are prepared using multiple layers of laminas in different directions, which means that the mechanical properties of laminates can be designed flexibly for practical applications. Distinct from isotropic materials, such as metals, the failure mechanism and failure mode are more complex due to the anisotropy of laminated composite materials [7,8]. When dealing with angle-ply laminates, the mismatch of the elastic moduli between adjacent layers can result in inter-laminar stresses at the free edge of the laminates [9,10]. Furthermore, the inter-laminar performance of FRP composite laminates is relatively weak, so higher inter-laminar stress may lead to the failure mode of delamination. Even a low stress level will cause fatigue delamination damage (FDD), as the performance of the inter-laminar material degrades [11]. In addition, the issue of FDD is more serious and critical in fiber metal laminates (FMLs) with openings [12]. Therefore, it is desirable to find a universal model of FDD for different composite laminated structures that can simply and efficiently evaluate the fatigue life span of delamination.

Based on a review of a large number of studies, fatigue delamination damage models (FDDMs) of composite laminated structures can be divided into the strength model based on stress–strain field analysis [13,14], the fracture mechanics model [15,16], the damage mechanics model [17,18,19], and the hybrid model [20,21,22,23], according to their theoretical frameworks. In general, the performance of a FDDM can be verified via delamination experiments, such as the double cantilever beam (DCB) [24], mixed-mode bending (MMB) [25], and the single-lap joint (SLJ) [26]. At present, the Paris law [27] for metals based on fracture mechanics has been commonly used to characterize the fatigue delamination growth (FDG) in the FDDMs of composite materials. The rate of crack propagation in fatigue is usually described by the Paris law. The simplest formula of the Paris law can be expressed as: d*a*/d*N* = *C*(*K_max_*)*^m^*, which relates the rate of crack propagation and the stress intensity factor (SIF). When the Paris law is applied to metals and composites, the largest difference lies in the characterization of crack growth. The former is characterized by the SIF [28], while the latter is characterized by the strain energy release rate (SERR) [29]. Considering the complexity of composite laminated structures and loading conditions, various methods have been used to characterize phenomenological models. Based on the influence factors of FDD, researchers have proposed a series of empirical and semi-empirical correction formulas [25,30,31], which provide methods of damage evolution for the simulation of FDDMs. In recent years, the physical model, a cohesive element based on damage mechanics [20,32,33], has received extensive attention and has been extensively researched. The cohesive element possesses a simple principle and high efficiency in numerical computation, which has the ability to easily obtain the FDG. In addition, several models in conjunction with the strength criterion with the fatigue progressive damage theory [34] have also been applied to estimate fatigue delamination propagation, which shows good agreement with experiments. Furthermore, investigators have already studied hybrid theoretical frameworks, which combine fracture mechanics and damage mechanics using chain rules [35]. The performances of these models have been verified via experiments and numerical simulations, and several practical conclusions have been also obtained. However, there are still many problems to be solved.

**Figure 1:**
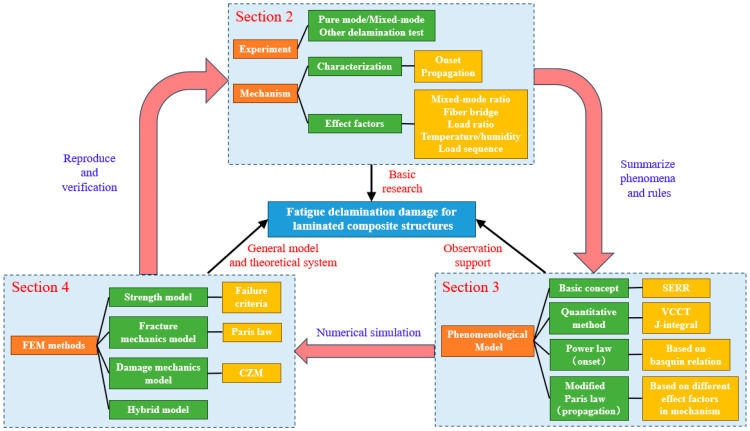
The guide map of the overall review on fatigue delamination damage for laminated composite structures.

Therefore, this review has been divided into five sections. Section 2 introduces the characterization methods and influence factors of fatigue delamination. In Section 3, the general formulas and procedures of typical phenomenological models have been summarized, including delamination onset and propagation under fatigue load. Based on the theoretical framework, Section 4 classifies the common methods of numerical simulation for fatigue delamination. Furthermore, the advantages and limitations of various FDDMs have been compared in detail. Finally, Section 5 sums up the up-to-date development of the FDDMs and provides suggestions on the research focuses of fatigue delamination. The overall framework of this review has been made into a guide map, as shown in Figure 1.

## 2. Experiment and Mechanism of Fatigue Delamination

In order to investigate the delamination of composite laminated structures under fatigue load, many researchers have carried out numerous experimental studies on quasi-static delamination. In order to quantitatively characterize FDD, the relationship between the crack growth rate (da/dN) and the fatigue load is established using fatigue crack growth methods for metal and experimental phenomena. However, the influence factors of fatigue delamination are various and complex. It is necessary to investigate the damage mechanism of fatigue delamination. In conclusion, studies on experimental phenomena and the delamination mechanism of composite laminated structures under fatigue load are significant. It is the foundation of the phenomenological model.

### 2.1. Delamination Test

**Figure 2:**
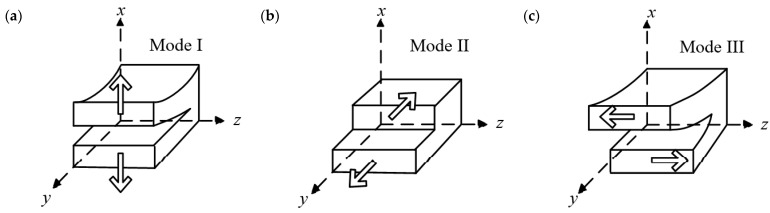
Different delamination modes in composite laminates. (**a**) Open mode. (**b**) Sliding mode. (**c**) Tearing mode.

According to the deformation characteristics of the crack tip, the mode of delamination crack growth for composite laminated structures can be divided into the open mode (mode I), the sliding mode (mode II), and the tearing mode (mode III) under the framework of fracture mechanics, as shown in Figure 2. Moreover, in order to characterize the relationship between fracture resistance and crack length, the resistance curve (R-curve) is commonly used to describe this phenomenon of interface resistance increase [36].

#### 2.1.1. Pure Mode Delamination Test

**Figure 3:**
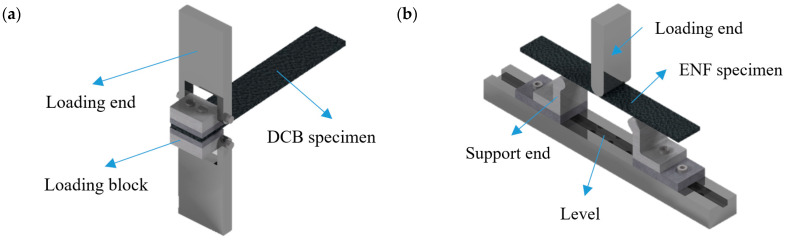
Delamination test bench for mode I and mode II. (**a**) DCB test. (**b**) 3ENF test.

In order to obtain mode I inter-laminar fracture toughness [37], ASTM has made a standard for the DCB test [38], with respect to unidirectional FRP composite laminates, as shown in Figure 3a. Due to the simple procedure, intuitive methods, and lower interference, the DCB test is one of the most commonly used tests for researchers to verify the proposed FDDMs.

ASTM has proposed a three-point bend end notched flexure (3ENF) test standard [39] for the mode II delamination test, as shown in Figure 3b. However, the delamination crack growth is very unstable, which results in an inaccurate evaluation of the initial SERR. Furthermore, the complete R-curve cannot be obtained, which exerts a significant impact on the input parameters of FDDMs [40,41]. Therefore, the test methods for mode II are still incomplete. In addition to 3ENF, other test methods, such as the center notched flexure (CNF) [42], the over notched flexure (ONF) [43], the four-point bend end notched flexure (4ENF) [44], and the end load split (ELS) [45], also have different limitations. When verifying the performance of FDDMs, it is required to select an appropriate test method.

There has been no recognized experimental standard for the mode III delamination test up to now. The experimental results obtained via edge crack torsion (ECT) [46] and split cantilever beam (SCB) [47] are sensitive to geometric parameters of structures. In addition, the results are affected by the mixed-mode ratio, which lead to inaccurate measurements. As the mode III delamination test is still an open investigation subject, it has been conservatively assumed that the delamination mechanism of mode III is similar to that of mode II [1]. However, the fracture toughness of mode III is slightly higher than that of mode II [48].

#### 2.1.2. Mixed-Mode Delamination Test

**Figure 4:**
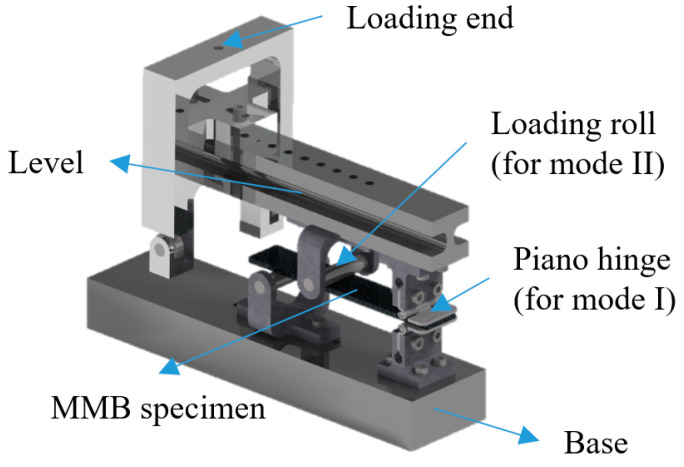
Delamination test bench for mixed-mode I/II (MMB test).

In general, composite laminated structures are under a mixed-mode load. Thus, the test methods for I/II mixed-mode delamination have been developed, including single leg bending (SLB) [49], fixed ratio mixed mode (FRMM) [50], cracked lap shear (CLS) [51], asymmetric double cantilever beam (ADCB) [52], and mixed-mode bending (MMB) [53]. It should be noticed that the MMB test could be converted into the DCB test and 3ENF test depending on the fixture location. Moreover, MMB has developed a specific analytical solution, which is included in ASTM [54], as shown in Figure 4.

#### 2.1.3. Other Delamination Tests

**Figure 5:**
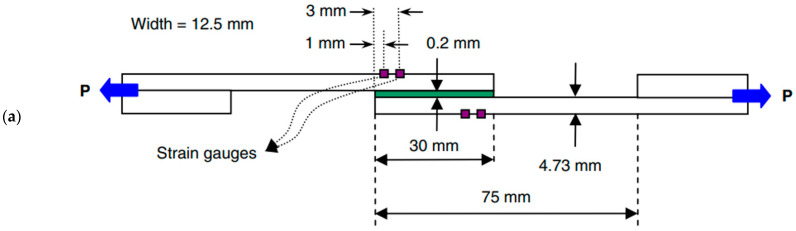

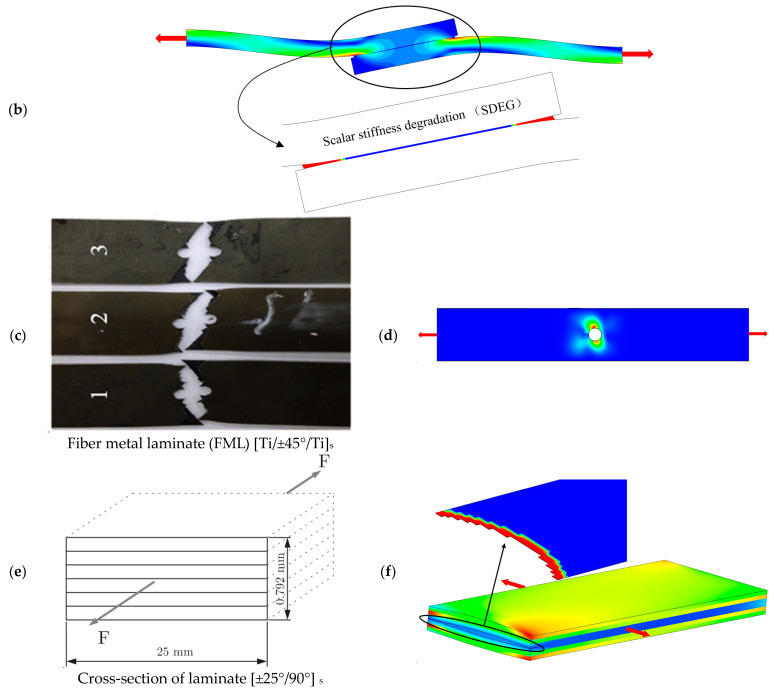
Other delamination test bench. (**a**) Schematic of single-lap joints [55]; (**b**) simulation of single-lap joints; (**c**) schematic of open-hole composite laminates [12]; (**d**) simulation of open-hole composite laminates; (**e**) schematic of an angle-laid composite laminate [57]; and (**f**) simulation of an angle-laid composite laminate.

In engineering, the stacking sequence and ply orientation of composite laminated structures are complex. Thus, investigators often carry out fatigue delamination tests on the following composite laminated structures in order to verify the universality of FDDMs. Firstly, fatigue delamination tests can be studied using the single-lap joints (SLJs) [55] and the double-lapped joint (DLJ) [56]. Second, fatigue delamination tests of open-hole composite laminated structures [12] with different diameters and locations have also been explored. Third, free-edge delamination tests for angle-ply composite laminates [57] have been further investigated. Figure 5a,c,e show the schematics of these three types of composite laminated structures. What is more, Figure 5b,d,f present the corresponding finite element model simulations independently finished by the authors of this review for comparison. 

### 2.2. Characterization for Fatigue Delamination 

Based on the above delamination tests, the delamination mechanism of composite laminated structures is gradually revealed. By summarizing the experimental phenomena, researchers have obtained many characterization methods for the fatigue delamination of FRP composites, which include fatigue delamination onset and propagation stages. Unlike metal materials, which have an obvious crack nucleation stage, the delamination onset of composite materials is difficult to detect. Various initial damage modes, such as voids, wrinkles, and delamination, are unavoidable. What is more, the anisotropy of the composite materials also makes delamination propagation obscure. Therefore, this review focused on characterizing the fatigue delamination onset and propagation of composite laminated structures under fatigue loads according to experimental phenomena.

#### 2.2.1. Fatigue Delamination Onset

**Figure 6:**
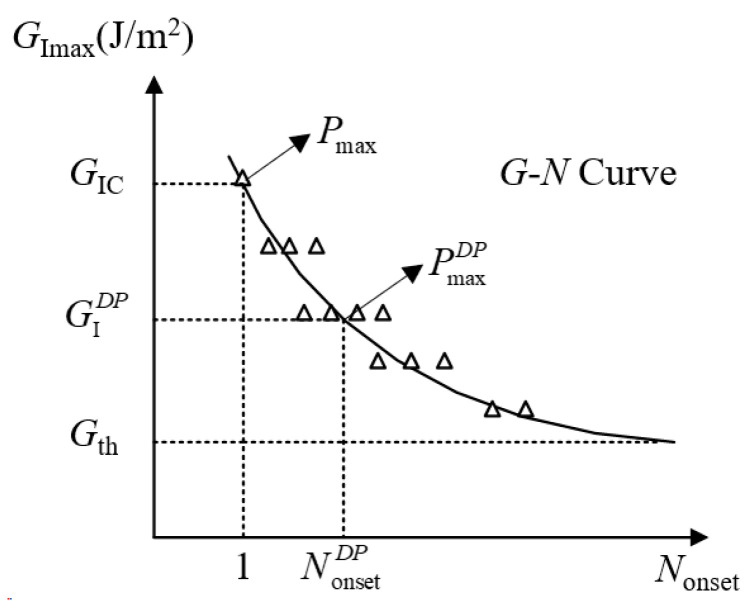
Typical onset curve of fatigue delamination.

Firstly, the mode I fatigue delamination onset of FRP composites are considered [58]. The delamination onset is determined by monitoring the crack tip or flexibility of DCB specimens. The purpose of this test is to establish the relationship between the maximum cyclic SERR (*G*_Imax_) and the number of cycles to delamination onset (*N*_onset_). What is more, the G–N curve is similar to the S–N curve, which is commonly applied in metal fatigue. The critical fracture toughness (*G*_IC_) is equivalent to the static strength (*σ*_s_), and the fatigue SERR threshold (*G*_th_) is equivalent to the fatigue strength limit (*σ*_f_). The simplest power law function for the G–N curve is expressed as Equation (1), where *A* and *m* are experimental fitting parameters. The formula for calculating the SERR G is also given in Equation (1), where *U* denotes the total elastic strain energy of the test specimen determined via fatigue load, and *b* and *a* represent the specimen width and delamination length, respectively. A typical fatigue delamination onset curve [58] is shown in Figure 6, where the superscript ‘*DP*’ represents the ‘percentage for maximum cyclic load *P*_max_ at *N*_onset_ = 1 to decrease by’. For example, when *DP* = 10%, $P_{\max}^{10\%}=0.9P_{\max}$. $N_{\mathrm{onset}}^{10\%}$ means the number of fatigue cycles for $P_{\max}^{10\%}$, and $G_{I}^{10\%}$ denotes the open mode I SERR corresponding to $P_{\max}^{10\%}$.

(1) $$G_{\mathrm{Imax}}=A{\left(N_{\mathrm{onset}}\right)}^{-m}\;G=-\frac{1}{b}\frac{dU}{da}$$

#### 2.2.2. Fatigue Delamination Propagation

**Figure 7:**
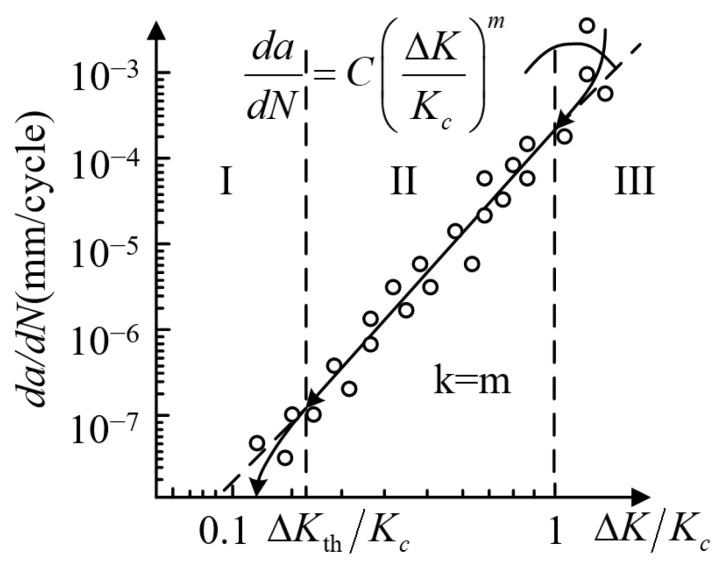
Typical normalized fatigue delamination crack growth curve.

During the FDG test, the delamination length (*a*) and fatigue cycle (*N*) are under a whole-process monitor. The experimental data are utilized to fit the relationship between crack growth rate and fatigue load range. Furthermore, the expression of fatigue delamination propagation adopts the general formula of the classical Paris law, like Equation (2), where *K* and *G* denote the SIF and the SERR, respectively, and *C* and *m* represent experimental fitting parameters. Therefore, the typical curve of normalized fatigue delamination crack growth (*da/dN* − ∆*K/K_c_*) [27] is shown in Figure 7.

(2) $$da/dN=C\times {\left[f(G\;\mathrm{or}\;K)\right]}^{m}$$

Similar to fatigue delamination onset, mode I delamination is selected as an example. As shown in Figure 7, the fatigue crack growth rate develops from the high-rate region III to the low-rate region I, which is following the direction of the arrow. As the cracks propagate along the fiber direction, the load range of the crack tip and the fatigue crack growth rate gradually decrease [59]. Moreover, the typical function of *f*(*K*) = ∆*K/K_c_* in Figure 7 can be replaced with another expression, such as the SIF range (∆*K*) [60] and SERR range (∆*G*) [29]. 

In medium-rate region II (Paris law region), the crack growth rate is linearly logarithmic with the load range. Then, the propagation of the crack stops when reaching the lower limit of fatigue delamination load range (∆*K*_th_). The fatigue crack growth rate of FRP composites is measured according to the ASTM standard of metal crack growth [61]. It is worth noting that the slope of the fatigue crack growth rate curve of most FRP composites is much larger than that of metals [62]. Therefore, it has been indicated that a little disturbance of the load range can produce large errors in the fatigue crack growth rate. In other words, the variation of the fatigue crack growth rate of composites is more sensitive than that of metals.

### 2.3. Influencing Factors of Fatigue Delamination

The relationship between the fatigue crack growth rate and the load range is usually used to characterize fatigue delamination propagation. Thus, the parameters involved in the load range [63,64,65,66] and different forms of load range [29] may directly influence FDG. These main influencing factors include the mixed-mode ratio, fiber bridging, load ratio, temperature [31,67], humidity [68], load sequence [65], and expression of *f* (*K or G*) in Equation (3). Moreover, there are several secondary factors, such as load frequency and wave form.

#### 2.3.1. The Mixed-Mode Ratio

**Figure 8:**
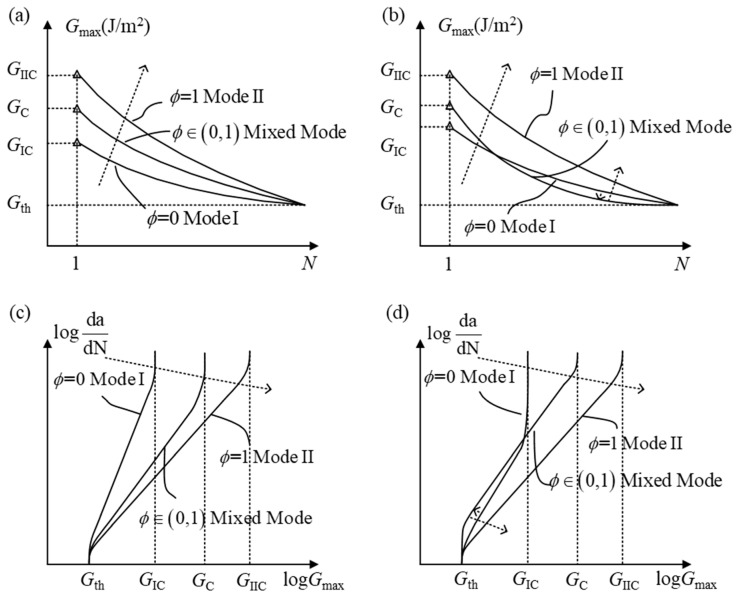
Influence of ϕ on typical fatigue delamination onset/propagation curves [1]. (**a**) The influence of ϕ on the $G_{\max}-N$ curve under a monotonic function. (**b**) The influence of ϕ on the $G_{\max}-N$ curve under a monotonic function. (**c**) The influence of ϕ on the $\log\left(da/dN\right)-\log\left(G_{\max}\right)$ curve under a non-monotonic function. (**d**) The influence of ϕ on the $\log\left(da/dN\right)-\log\left(G_{\max}\right)$ curve under a non-monotonic function.

According to the definition of the mixed-mode ratio in terms of energy, the mixed-mode ratio is: *ϕ = G*_II_*/G* (*G* = *G*_I_ + *G*_II_). In general, *G*_IIC_ is obviously greater than *G*_IC_ in FRP composites [25]. As shown in Figure 8a,c, *ϕ* ranges from zero to one with the transition from mode I to mode II. In addition, the fracture toughness of the mixed mode changes from *G*_IC_ to *G*_IIC_. It can be found that the upper limits of *G*_max_–*N* curves and log(*da/dN*) − log(*G*_max_) curves changed with different *ϕ*. However, the lower limits of the SERR remain unchanged. In other words, the mixed-mode ratio directly affects the delamination onset and fatigue crack growth rate [69]. Nevertheless, the influence of the mixed-mode ratio can be ignored under a low fatigue crack growth rate. These rules have been confirmed through experimental investigations conducted by Obrien [70]. 

In order to accurately describe the relationship between the delamination onset/fatigue crack growth rate and the mixed-mode ratio, researchers [67,71] have proposed a general curve fitting model based on a series of fatigue delamination tests of FRP composites. It is the most important that the fitting coefficient is a non-monotonic function of *ϕ*, which makes the prediction of delamination onset and propagation more precise. As shown in Figure 8b,d, the changes in the maximum SERR (*G*_max_) and fatigue crack growth rate (*da/dN*) are divided into two stages. In the first stage, *G*_max_ and *da/dN* monotonically change with the increase in the mixed-mode ratio. In the second stage, *G*_max_ and *da/dN* non-monotonically change along the arrow’s direction as the mixed-mode ratio increases. The experimental rules on the mixed-mode ratio mentioned above have been depicted by Bak et al. [1], which has been recovered in Figure 8.

#### 2.3.2. Fiber Bridging Effect

In test standards, most test objects of fracture toughness and fatigue crack growth are unidirectional FRP composite laminates. The influence of fiber bridging [72] on the critical SERR (*G*_C_) is artificially introduced, which can obtain a complete R-curve [73,74]. From the initial stage of a fiber bridging region [75], the critical SERR increases and eventually tends to a stable value. Research conducted by Zhang et al. [66] has shown that the fiber bridging effect will lead to a more complex characterization of delamination onset and propagation. This means that the lower limit (*G*_th_) of curves will depend on the mixed-mode ratio. 

**Figure 9:**
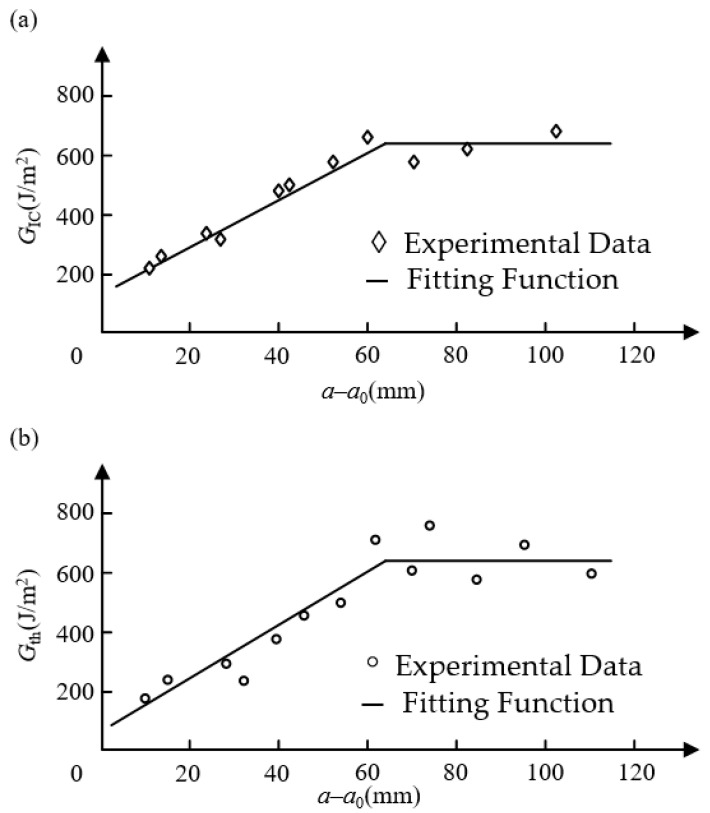
Effect of fiber bridging on inter-laminar performance [76]. (**a**) Influence of fiber bridging on $G_{\mathrm{IC}}$. (**b**) Influence of fiber bridging on $G_{\mathrm{th}}$.

As shown in Figure 9a,b, Yao et al. [76] carried out the mode I fatigue delamination test and fitted curves of the *G*_IC_ and *G*_th_ with delamination length. The laminates were produced by hand-lay-up of 32 thermosetting unidirectional carbon/epoxy prepreg layers of M30SC/DT120. It can be found that the fracture toughness of mode I (*G*_IC_) and lower limit of the SERR (*G*_th_) can be related to a fatigue crack length of *a*–*a*_0_ via piecewise functions. *G*_IC_ and *G*_th_ increase to stable values, which have a significant promotion when compared with the initial values. If the influence of fiber bridging is neglected, and the stable values of inter-laminar parameters are taken for numerical simulation, it will lead to a conservative estimation of fatigue delamination life for FRP composites.

#### 2.3.3. The Load Ratio

**Figure 10:**
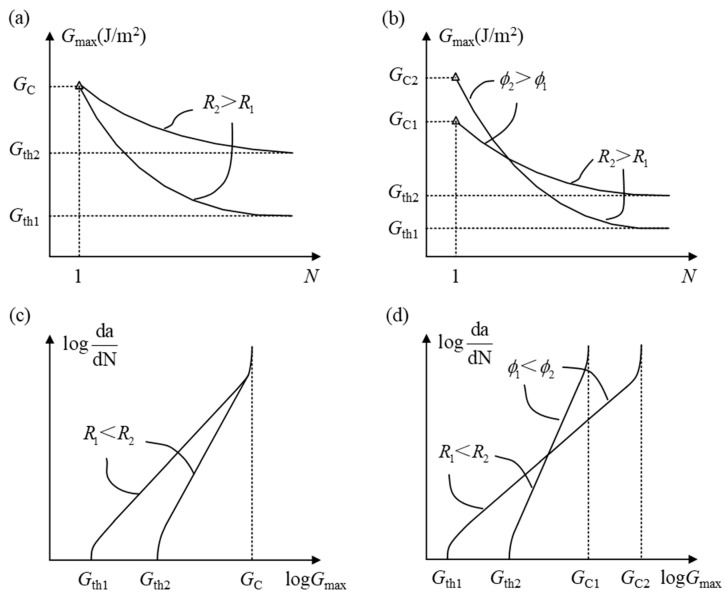
Effects of the load ratio (R) on the delamination onset and propagation curves [1]. (**a**) Influence of R on the delamination onset curve. (**b**) Coupling influence of R and ϕ on the delamination onset curve. (**c**) Influence of R on the delamination propagation curve. (**d**) Coupling influence of R and ϕ on the delamination propagation curve.

When studying fatigue delamination in composite materials, the load ratio (*R*) has always been the focus. *R* equals to the ratio of maximum and minimum fatigue load (*P*_min_/*P*_max_), where *P*_min_ and *P*_max_ determine the maximum and minimum SERR (*G*_max_ and *G*_min_), respectively. If two of three load parameters (*G*_max_, *G*_min_, and *R*) are known, the form of fatigue load can be fixed. Therefore, while *G*_max_ is fixed, the effect of the load ratio on delamination onset and propagation is investigated. As shown in Figure 10a,c, the experimental study of Matsubara et al. [77] indicated that the higher the load ratio, the higher the lower limit of the delamination onset curve, which increases the number of cycles for delamination onset. Meanwhile, the larger the average load, the greater the slope of the delamination propagation curve, which increases the sensitivity of the fatigue crack growth rate to *G*_max_. 

In order to simultaneously consider the effects of the mixed-mode ratio and load ratio, Allegri et al. [78] attempted to establish a relationship between coupling influence curves in the form of Figure 10b,d. In the initial phase of the fatigue load, the higher the mixed-mode ratio, the greater the maximum SERR. When the propagation of fatigue delamination occurs, the higher the load ratio, and the greater the maximum SERR under the same fatigue crack growth rate. Therefore, when considering the coupling effects of the mixed-mode ratio and load ratio, the contribution of both to fatigue delamination onset and propagation must be reasonably allocated. It is significant to obtain a phenomenological model via experiments. The experimental observations on delamination onset and propagation curves have been summarized by Bak et al. [1] in Figure 10.

#### 2.3.4. Other Major Factors

Due to long-term service under hygrothermal conditions, the delamination resistance of composite laminates is weakened. The tests of Springer [79], Jin et al. [31], and Ramirez et al. [80] showed that the performance of the polymer matrix will be influenced under high temperature and humidity, especially for FRP composites. Therefore, the inter-laminar properties of composite laminates will be directly affected. Hence, it is necessary to take temperature and humidity as parameters, which influence the fatigue crack growth rate in special environments. Based on experiments, the quantitative expressions of fatigue delamination behavior are obtained in corresponding material system and load condition.

There is no consensus on the mechanism and effect of fatigue load sequence for delamination propagation. On the basis of micromechanics, Li [81] have tried to study the interfacial fatigue debonding of unidirectional ceramic matrix composites under different load sequences. In addition, Van Paepem and Degrieck [65] reviewed analytical methods of high–low fatigue load and low–high fatigue load of FRP composites. Thus, several issues and ideas on influences of load sequence for fatigue delamination have been proposed.

When the Paris law is extended from metal to composite materials, the problem of stress singularity at the crack tip is inevitable. Therefore, in the theoretical framework of linear elastic fracture mechanics (LEFM), the SERR (*G*) is established from the perspective of energy. In addition, the relationship between the SERR (*G*) and the SIF (*K*) is derived, which realizes the substitution of the load range. After fitting the experimental data, there are various functions, such as ∆*G*, *G*_max_, $\sqrt{G_{\max}}$,${\left(\sqrt{G_{\max}}-\sqrt{G_{\max}}\right)}^{2}$, and so on [29,82,83], which have different intrinsic physical meanings.

#### 2.3.5. Other Secondary Factors

In general, loading frequency [65,84] and loading rate [85] are secondary factors in determining strain energy release rate. As long as the frequency is below 10 Hz, the limitation of frequency ensures that composite materials do not produce local high temperatures. Thus, the fatigue crack growth rate will not be affected. The influence of load waveform is much smaller [84], so researchers have rarely investigated on it. 

## 3. Phenomenological Model of Fatigue Delamination 

Referring to Section 2, the analytical methods of fatigue delamination are established. Based on qualitative analysis of the experimental phenomenon, the significant factors affecting fatigue delamination onset and propagation have been clarified. In this section, the calculation of the SERR is simply presented. Then, from the different perspectives of influence factor and data processing, a number of phenomenological models are introduced in detail.

### 3.1. Calculation of the SERR

Analytical solutions and the finite element method (FEM) are commonly employed to solve the SERR. The specific methods of analytical solution mainly include modified beam theory (MBT), modified compliance calibration (MCC), and so on [38,39,54]. In addition, the area method can also roughly estimate the SERR [86,87]. The FEM methods include the virtual crack closure technique (VCCT), the J-integral method, the virtual crack extension, and so on. Among them, the VCCT and the J-integral method are most widely used.

#### 3.1.1. The VCCT

**Figure 11:**
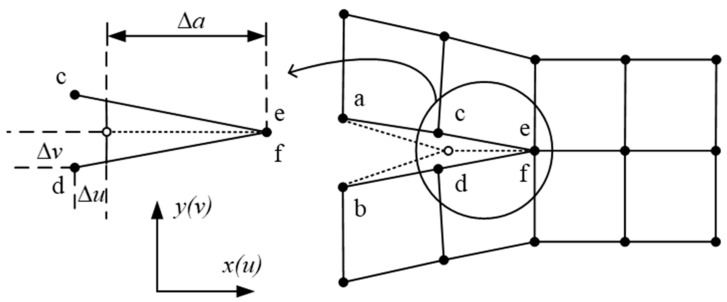
The schematic diagram of calculating the SERR using the VCCT.

The VCCT has been rapidly developed since Rybicki and Kanninen [28] proposed it in 1977. The basic assumption of the VCCT [75] is that the opening displacement of the virtual crack tip is approximately equal to that of the initial crack tip. In the two-dimensional (2D) FEM shown in Figure 11, the SERRs of pure modes I and mode II of delamination are calculated using Equation (3):

(3) $$\begin{array}{l} G_{I}=\frac{1}{2B\Delta a}F_{y}\Delta v\\ G_{II}=\frac{1}{2B\Delta a}F_{x}\Delta u \end{array}$$

where *B* is the crack thickness, ∆*a* is the crack length, *F_x_* and *F_y_* denote the forces of node e and node f along the *x* and *y* directions, respectively, and ∆*u* and ∆*v* denote the nodal displacement difference between node c and node d along the *x* and *y* directions, respectively.

However, the damage evolution path of the VCCT needs to be set up in advance. Without the prefabricated cracks, the delamination evolution path cannot be determined in structures with complex geometric characteristics and load conditions. Moreover, the VCCT can only track delamination propagation but cannot predict delamination onset [6].

#### 3.1.2. The J-Integral Method

**Figure 12:**
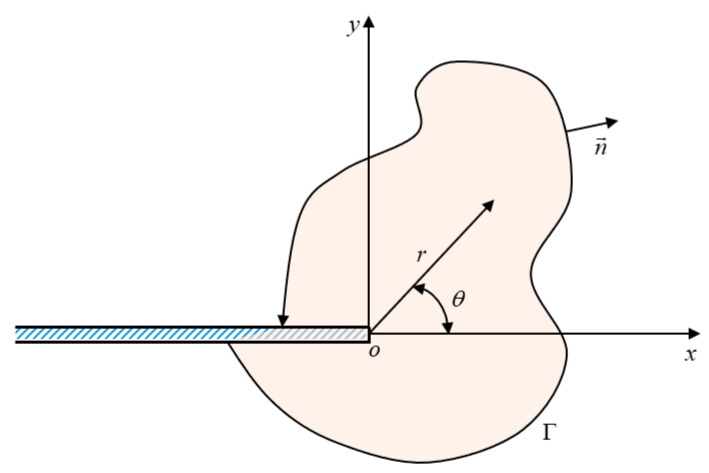
Typical coordinates for the description of path-independent J-integrals [88].

In 1968, Rice and Rosengren [88] proposed the J-integral method for 2D problems. The far-field stress and displacement are solved using line integrals, which are used to describe the mechanical properties of the crack tip. Since the phenomenological models involved in this review are all based on the theoretical framework of LEFM, the J-integral and SERR are completely equivalent for problems of plane stress or plane strain. Therefore, the SERR can be easily calculated via a line integral, which is independent with the path from the lower surface to the upper surface of the crack. The schematic of typical coordinates for the description of path-independent J-integrals is shown in Figure 12, where *o* denotes the crack tip, Γ denotes the selected random contour of the path for the J-integral, and $\vec{n}$ denotes the normal vector of contour Γ. In addition, the path-independent is traversed in the contra-clockwise sense. The specific formula of the J-integral is presented in Equation (4):

(4) $$\begin{array}{l} J=\int_{\Gamma }\left[W\left(\varepsilon \right)dy-T\cdot \frac{\partial u}{\partial x}ds\right]\\ W\left(\varepsilon \right)=\int_{0}^{\varepsilon }\sigma_{ij}d\varepsilon_{ij},\;T_{i}=\sigma_{ij}n_{j} \end{array}$$

where *W* denotes the energy density of an elastic material, *T* is the traction vector on contour Γ, *u* is the displacement vector, and *s* denotes the arc length. 

### 3.2. Delamination Onset Phenomenological Model

The simplest power law function, like Equation (1), is applied to fit experimental data on delamination onset for FRP composites, which is based on the Basquin relation [89]. However, the model does not consider the main influencing parameters, such as the mixed-mode ratio, loading ratio, and temperature. Any change in the structure, environment, and load condition will result in different curve fitting results. Thus, an easy formula like Equation (1) has poor universality in practice.

Jagannahan et al. [90] introduced the concept of the constant life diagram (CLD), which is commonly used in metal fatigue. On this basis, the constant onset life diagram (COLD) of mode II fatigue delamination of carbon fiber-reinforced polymer (CFRP) composites was established. After fitting the test data of three load ratios, *R* = 0, *R* = 0.5, and *R* = 1 (that is pure mode II), using the Basquin formula [89], the curve between the amplitude of the SERR and the average SERR (*G*_a_–*G*_m_ curve) was achieved. The COLD is obtained by connecting points of constant life. Then, the number of cycles of fatigue delamination onset under the corresponding load level can be obtained via linear interpolation.

However, regarding the latest research on mode I and mode II fatigue delamination onset of CFRP materials in hygrothermal conditions, Ramirez et al. [80] still adopted the same model as Equation (1). It is obvious that there is no general model that can comprehensively consider influence factors of fatigue delamination onset. There are few phenomenological models [1,2,58,80,90] for fatigue delamination onset in composite materials, and there is a lack of experimental verification.

### 3.3. Delamination Propagation Phenomenological Model

Up to now, the phenomenological models of fatigue delamination propagation in composite materials are mostly based on the classical Paris law. According to the discussion on influence factors of the fatigue crack growth rate in Section 2.3, the commonly modified Paris law model is unified and summarized. The main influencing factors include the mixed-model ratio, fiber bridge, load ratio, and temperature, which will be discussed in detail. Secondary influencing factors include load sequence [65,81,84,91], load frequency, and load waveform [92], which are less studied, so this paper will not elaborate on them. The original formula of the modified Paris law model is as follows:

(5) $$\frac{da}{dN}=C\;f{\left(K\;or\;G\right)}^{m}$$

#### 3.3.1. Mixed-Mode Ratio Modified Model

For the Paris law considering the mixed-mode ratio, the SERR is generally used to characterize the load level of composite materials. Thus, the similar parameters *f* (*K* or *G*) in Equation (5) are simplified to *f* (*G*). When mode III is assumed to be similar to mode II, four issues need to be considered while establishing the mixed-mode ratio modified model. 

Firstly, it needs to determine how to define the contribution of different modes to the fatigue crack growth rate under mixed-mode loads. Therefore, uncoupled models [93,94] and coupled models [95] will be proposed. Secondly, it requires to determine the approach of normalizing the load level [79,96]. Thirdly, coefficients of the Paris law can be set as material constants or functions of the mixed-mode ratio. If functions of the mixed-mode ratio are adopted, monotonic [97,98] or non-monotonic [25] function relationships will be presented. Fourthly, an approach introducing the upper and lower limits [99] of the fatigue crack growth rate to the Paris law model is also required.

Based on the simplest uncoupled models of mode I and mode II, a modified Paris law that considers an uncoupled mixed-mode can be proposed via linear superposition. The general formula is given by:

(6) $$\frac{da}{dN}=\sum_{i}C_{i}f{\left(G_{i}\right)}^{m_{i}}\;\;\;\;i=I,II$$

where *C_i_* and *m_i_* are the material constants of mode *i*, and *f* (*G_i_*) denote the functions of load levels. Different models can be obtained using different functions. For example, the models of Gustafson and Hojo [94] can be obtained when *f* (*G_i_*) = *G_i_*_max_ − *G_i_*_min_. Nevertheless, the models of Ramkumar and Whitcomb [93] can be obtained when *f* (*G_i_*) = *G_i_*/*G_ic_*, which is normalized by the critical SERR of mode *i*. Therefore, the fatigue crack growth rates of different FRP composites can be compared.

However, composite laminated structures under fatigue load are usually in mixed mode. What is more, the interaction between mode I and mode II/III in delamination propagation cannot be ignored. The simplest general interaction model between mode I and mode II is as follows:

(7) $$\frac{da}{dN}=C(\phi )f{\left(G\right)}^{m(\phi )}$$

When considering the general linear interaction:

(8) $$\begin{array}{l} C(\phi )=\sum_{i}C_{i}\phi_{i},\;\sum_{i}\phi_{i}=1\\ m(\phi )=\sum_{i}m_{i}\phi_{i},\;\sum_{i}\phi_{i}=1 \end{array}$$

It is observed that the essence of the coupling model is to regard the coefficients of the Paris law (*C* and *m*) as a function of the mixed-mode ratio (*ϕ*). However, *C*(*ϕ*) and *m*(*ϕ*) in Equation (7), which follow the linear coupling relationship like Equation (8), are monotonic functions. For the epoxy matrix composite, it has been proven [96] that the coefficients of the Paris law are non-monotonic functions of the mixed-mode ratio. Therefore, a non-monotonic function can be constructed, which needs to satisfy two conditions. Firstly, the values of the endpoint must be equal to the Paris law coefficients of mode I and mode II. Secondly, the stationary point of the non-monotonic function must be within the range between zero and one. The quadratic function *ϕ* can satisfy requirements by controlling boundary conditions:

(9) $$\begin{array}{l} m(\phi )=d+e\phi +f\phi^{2},\;m(0)=m_{I},\;m(1)=m_{II}\\ C(\phi )=a+b\phi +c\phi^{2},\;C(0)=C_{I},\;C(1)=C_{II} \end{array}$$

The most general non-monotonic modified model, including the effect of the mixed-mode ratio, was proposed by Blanco et al. [25]. This model has been established via experimental observations in Section 2 and the general construction method mentioned above.

As the formula and region II in Figure 7 presents, the classical Paris law only describes the middle-rate region of fatigue delamination. In order to simultaneously describe the thresholds of the fatigue crack growth rate curve, it is possible to multiply Equation (7) by functions that represent the lower and upper horizontal asymptotes. The general formula for this kind of model is given as follows:

(10) $$\frac{da}{dN}=C(\phi )f{\left(G\right)}^{m(\phi )}g_{th}(G)h_{c}(G)$$

where the load level *G* can be expressed in various forms and normalized or not. The multiplier *g*_th_(G) characterizes the lower horizontal asymptote of the fatigue crack growth rate curve. This function needs to satisfy two conditions. When the load level is infinitely close to the fatigue crack growth threshold (*G*_th_), the value of the function tends to be zero. When the load level is slightly larger than the fatigue crack growth threshold (*G*_th_), the value of the function tends to be one. In a similar way, the multiplier *h*_c_(G) characterizes the upper horizontal asymptote of the fatigue crack growth rate curve. This function needs to satisfy two conditions. When the load level is infinitely close to the critical fatigue crack growth (*G*_c_), the value of the function tends to be positive infinite. When the load level is slightly less than critical fatigue crack growth (*G*_c_), the value of function tends to be one.

Finally, the requirements of *g*_th_(*G*) and *h*_c_(*G*) are satisfied simultaneously. Therefore, the modified Paris law that can describe the complete fatigue crack growth rate curve is obtained. The classical functions *g*_th_(*G*) and *h*_c_(*G*) can refer to the phenomenological model of Shivakumar et al. [99].

#### 3.3.2. Fiber Bridge-Modified Model

Based on the extensive application of unidirectional composite laminates in practice, the influence of fiber bridges on fatigue crack growth rate cannot be neglected. By observing the phenomenological models that consider the fiber bridge effect based on the Paris law, four issues need to be solved when establishing the model. 

Firstly, it needs to select a proper parameter which is similar to *f* (*G*). Secondly, the methods considering the effect of the fiber bridge by normalization are required. Thirdly, it requires to be determined that the coefficient of the Paris law is a material constant or a function of fatigue crack length. Fourthly, it needs to present a method that introduces the upper and lower thresholds of the fatigue crack growth curve. Based on the above considerations, the following general formula is given as follows:

(11) $$\frac{da}{dN}=C(a)f{\left(G(a),G_{CR}(a)\right)}^{m(a)}$$

where *c*(*a*) and *m*(*a*) are the coefficients of the Paris law that depend on the fatigue crack length. *G*_CR_(*a*) denotes the *R*-curve obtained from the fracture toughness experiment. 

In the model of Shivakumar et al. [99], the definition of fatigue crack growth rate is simple. The functions *f = G*_max_(*a*)/*G*_CR_(*a*), *C*, and *m* denote the material constants. However, Gong et al. [83] deduced a reasonable form of the SERR via the superposition principle from the concept of the SERR. ${\left(\sqrt{G_{max}}-\sqrt{G_{min}}\right)}^{2}$ has a good agreement with the similar parameters ∆*K* in classical Paris law. Yao et al. [36,82] elaborated on how to consider the effect of fiber bridges on mode I fatigue crack growth rate in a modified Paris law. On the basis of the above similar parameters, the Hartman–Schijve equation [76,100] and the idea of an equivalent SERR at the crack tip [101] are used. Therefore, similar parameters that can consider the upper and lower thresholds of the fatigue crack growth curve and fiber bridge are, respectively, constructed by Equation (12). These two phenomenological models based on a modified Paris law have been verified via fatigue delamination tests. In addition, it has been concluded that the Paris law coefficient *m* of mode I fatigue delamination is independent of fatigue crack length in FRP composite materials:

(12) $$\begin{array}{c} f\left(G(a),G_{CR}(a)\right)=\frac{\sqrt{\Delta G(a)}-\sqrt{\Delta G_{th}(a)}}{\sqrt{\left[1-\sqrt{G_{\max}}(a)/\sqrt{G_{CR}(a)}\right]}}\\ f\left(G(a),G_{CR}(a)\right)=\frac{G_{0}}{G_{\mathrm{CR}}(a)}\Delta G(a) \end{array}$$

where ∆*G*(*a*), ∆*G*_th_(*a*), and *G*_max_(*a*) are functions of the SERR range, the fatigue crack growth threshold range, and the maximum SERR, which vary with the fatigue crack length *a*, respectively. *G*_0_ denotes the initial SERR without the effect of the fiber bridge. This model can well capture the fatigue crack growth rate at different fatigue crack lengths with high accuracy and simple fitting.

#### 3.3.3. Load Ratio-Modified Model

The load ratio of fatigue delamination has always been a focus. From the analysis in Section 2.3, the influence of the load ratio can be taken into consideration in a modified model by introducing the load level. The load level parameters include *G*_mean_, ∆*G*, and *G*_min_/*G*_max_. However, the addition of influencing factors to the model is not arbitrary. Therefore, five issues need to be concentrated on when establishing a general modified model. 

Firstly, it needs to select similar parameters in classical Paris law. Secondly, the method of normalization is required to be determined. Thirdly, it is required to build the expression of the load level. Fourthly, the coefficient of the Paris law may use a material constant or the function of the load ratio. Finally, an approach introducing the upper and lower thresholds of the modified Paris law is also required. Therefore, the general formula for developing such a model is as follows:

(13) $$\frac{da}{dN}=C(R)f{\left(G_{\max},G_{\min}\right)}^{m(R)}$$

where *C*(*R*) and *m*(*R*) are coefficients of the Paris law that depend on the load ratio. In the simplified model, *C* and *m* can also represent material constants. *f* (*G*_max_, *G*_min_) denotes all combined expressions of maximum and minimum load levels, including normalized models. The simplest expression of *f* (*G*_max_, *G*_min_) is *f* = *G*_max_ − *G*_min_.

Considering the load ratio in fatigue crack growth rate, the model proposed by Hojo et al. [63] is most commonly used. This model represents the relative contribution of the SIF range (∆*K*) and maximum SIF (*K*_max_) by parameter *γ*, which ranges from zero to one. The construction of function *f* (*G*_max_, *G*_min_) is shown in Equation (14):

(14) $$f\left(G_{\max},G_{\min}\right)=K_{\max}^{\gamma }\Delta K^{1-\gamma }$$

In fact, after taking the logarithm on both sides of Equation (14):

(15) $$\log f=\gamma \log K_{\max}+(1-\gamma )\log\Delta K$$

The kernel of constructing the function *f* (*G*_max_, *G*_min_) is the logarithmic weighted average of ∆*K* and *K*_max_. In fact, this concept is reflected in many classically modified Paris laws considering the load ratio. For instance, the expression of Equation (16) was specially proposed for glass fiber-reinforced laminated composites by Atodaria et al. [102].

(16) $$f={\left(\sqrt{G}\right)}_{\mathrm{mean}}^{\gamma }{\left(\Delta \sqrt{G}\right)}^{1-\gamma }$$

However, the models mentioned above do not consider the effects of high-rate and low-rate regions of the fatigue crack growth curve. By directly incorporating the horizontal asymptote function into *f* (*G*_max_, *G*_min_) by referring to Equation (10), the models of Shintarou et al. [103] consider the low-rate regions and that of Blanco et al. [25] consider both the high-rate and low-rate regions. The specific functions are given as follows:

(17) $$\begin{array}{c} f\left(G_{\max},G_{\min}\right)=\Delta K-\Delta K_{th}\\ f\left(G_{\max},G_{\min}\right)=\frac{2K_{\mathrm{mean}}\left(\Delta K-\Delta K_{\mathrm{th}}\right)}{K_{C}^{2}-K_{\max^{2}}} \end{array}$$

In fact, the model which considers the fiber bridges proposed in references [76,101,104] also take the effect of the load ratio into account. In addition to replacing similar parameters with ${\left(\sqrt{G_{max}}-\sqrt{G_{min}}\right)}^{2}$, the coefficient of the Paris law (*C* (*a*, *R*)) is set as a function of fatigue crack length (*a*) and load ratio (*R*). However, *m*(*R*) only depends on the load ratio (*R*). In this way, the fatigue crack growth curves under different load ratios are unified under the influence of fiber bridges.

#### 3.3.4. Temperature-Modified Model

As for the influence of hygrothermal conditions on the fatigue crack growth rate, the influence of temperature has been simply discussed here. The specific methods of studying the effect of humidity can be followed using the method of studying temperature. Miura et al. [105] have studied I/III mixed-mode fatigue crack growth curves of woven glass/epoxy composite laminates at 295 K (room temperature), 77 K, and 4 K. Their results indicated that fatigue crack growth rates under three different temperatures satisfy *da*/*dN*_295K_ > *da*/*dN*_4K_ > *da*/*dN*_77K_. However, the modified Paris law is primitive, just like Equation (18):

(18) $$\frac{da}{dN}=C{\left(\Delta G_{T}\right)}^{m}$$

Above all, there is an issue with the similarity of ∆*G*_T_ and ∆*K*. Secondly, the coefficient of the Paris law is not considered as a function of temperature. Therefore, not only can the accuracy and application of the model not be guaranteed, but also the influence of environmental temperature under the same material system cannot be unified in a single fatigue crack growth curve. The model of Zhong et al. [106] also exhibits similar defects.

In their latest investigations, Jin et al. [31] measured the tensile–tensile fatigue crack growth rate of unidirectional and orthogonal Ti/CFRP laminates at 25 °C, 80 °C, 120 °C, and 150 °C. The fatigue crack length non-linearly increased as the temperature increased. Moreover, acceleration of fatigue crack growth is obvious during the temperature difference from 120 °C to 150 °C. Based on the concept of the SIF, the general formula of the modified Paris law which considers the effect of temperature is given by:

(19) $$\frac{da}{dN}=C(T){\left(\Delta K\right)}^{m(T)}$$

According to the experimental phenomena, the fitting functions that satisfy the requirement of non-linear monotonic behavior of *C*(*T*) and *m*(*T*) are as follows:

(20) $$\begin{array}{l} C(T)=C_{0}+C_{1}e^{D_{0}T}\\ m(T)=m_{0}+m_{1}e^{n_{0}T} \end{array}$$

where *C*_0_, *C*_1_, *D*_0_, *m*_0_, *m*_1_, and *n*_0_ are the fitting parameters of experimental data. When material systems and environmental conditions remain unchanged, this kind of model with the coefficient of Paris law as the temperature function is very effective. The most important thing is that the reasonable fitting function should be adapted through observation of the experiment.

## 4. Finite Element Simulation of Delamination Fatigue

In this section, FDDMs are classified into four categories based on FEM simulations of fatigue delamination onset and propagation under fatigue load, in which the theoretical frameworks include the strength model, fracture mechanics model, damage mechanics model, and hybrid model. In order to comprehensively compare existing FDDMs, establishment process, application scope, prediction ability, and limitations of various models are all taken into account.

### 4.1. Strength Model

Generally, the fatigue model of FRP composites can be classified into three types [107]: the model based on the S–N curve, the residual stiffness/strength model, and the progressive damage model. The similarity between these models is that they are based on classical strength theory and the strength failure criterion. A similar method can be used to establish FDDMs of composite laminated structures.

During the early stages, delamination simulations are mainly based on strength theory. After obtaining stress fields of composite laminated structures, delamination damage is calculated using the strength failure criterion expressed by stress components. Among the strength failure criteria of composites, criteria such as maximum stress, maximum strain, Tsai–Hill, Tsai–Wu, and Hoffman are commonly used in engineering. For example, Bernasconi et al. [13] directly verified the correlation between the maximum shear stress and the number of fatigue cycles. Thus, the *τ*–*N* curve, which was obtained through the SLJ test, provided a simple and practical criterion for bonding structures. 

However, these criteria are not able to consider failure modes of delamination separately. Hence, investigators have proposed many strength failure criteria for delamination, such as the Hashin criterion [108], Chang-chang criterion [109], Hou criterion [110], Zou criterion [111], and LaRC criterion [112], which are shown in Table 1. These criteria are usually expressed by quadratic polynomials. Therefore, the essence of these criteria is transforming complex stress states into equivalent stress states. In many cases, in order to reduce the effect of stress singularity around the crack tip, stress strength criteria are also rewritten as strain strength criteria.

In order to avoid fatigue strength failure in engineering, the maximum stress of the fatigue load is usually less than 50% of the ultimate strength for composite material (50% of the ultimate strength is generally smaller than the fatigue strength limit, which is considered to be approximately infinite fatigue life) [19,29,113]. However, it is unreasonable to assume that the structures will never suffer from fatigue strength failure. Thus, material properties such as Young’s modulus and strength should be degraded in numerical simulations. On the basis of the abovementioned facts, the residual stiffness/strength model and the fatigue progressive damage model (FPDM) [114] were proposed. It should be noted that these models essentially belong to the theoretical framework of damage mechanics. Therefore, it will be elaborated in Section 4.3.

### 4.2. Fracture Mechanics Model

It should be emphasized that the fracture mechanics models are commonly based on the theoretical framework of LEFM [115,116,117]. LEFM supposes that the complexity of the crack tip is not considered, and that the stress singularity of the crack tip is avoided. Instead, stress states around the crack tip (the material property is assumed to be linear elasticity [1,118,119]) are used to characterize the fracture behavior.

Compared with the strength criterion, the fracture mechanics model has developed a series of standard tests and formulas for solving the stress field around the crack tip under various crack forms. It is necessary to calculate the SIF (*K*) for developing the fracture criterion. The unit of SIF (*K*) is [N][L]^−3/2^ (N for newton and L for meter), which lacks explicit physical meaning. However, the SERR (*G*) defined by Irwin [120] represents the energy consumption per unit area of crack growth, and the unit of SERR (*G*) is [N][L]^−1^. Through the strict proof of equivalence between *G* and *K*, a fracture criterion can be automatically transformed into an energy criterion. For instance, the relationship between the SERR (*G*_I_) and the SIF (*K*_I_) of mode I delamination is shown in Equation (21). The load levels can be characterized by specific physical meaning during fatigue crack growth. The main numerical computation method can be referred to in the brief introduction and references in Section 3.1.

(21) $$G_{I}=\frac{K_{I}^{2}}{E_{1}}$$

**Figure 13:**
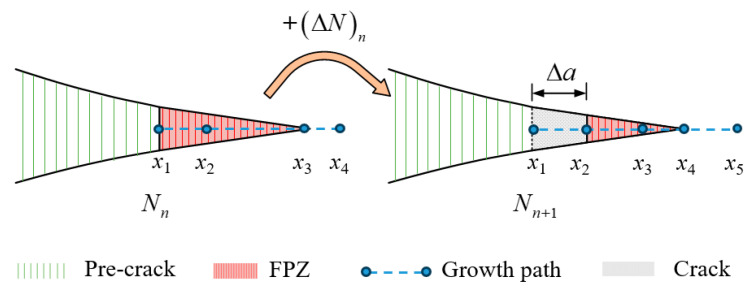
Fatigue crack growth model based on the Paris law of fracture mechanics.

In order to establish the relationship between the fatigue crack growth rate and the load level, almost all phenomenological models of fatigue delamination are based on the modified Paris law. Based on experiments, the influence of parameters and the construction of methods can be referred to in Section 2.2, Section 2.3, Section 3.2 and Section 3.3. The schematic diagram of FDDMs [121,122] based on the Paris law of fracture mechanics is shown in Figure 13. 

The green, red, and gray regions in Figure 13 denote the pre-crack, failure process zone (FPZ) [123], and crack. The blue dashed line represents the growth path of crack propagation. *N_n_* denotes the fatigue cycle number (*N*) of the *n* step, and *N_n_*_+1_ represents the fatigue cycle number (*N*) of the (*n* + 1) step. ${\left(\Delta N\right)}_{n}$ represents the fatigue cycle number between the *n* step and the (*n* + 1) step. Δa denotes the crack length during ${\left(\Delta N\right)}_{n}$. The steps, in detail, are as follows:

1. Artificial pre-cracks are added to composite laminated structures. Set the growth path of crack propagation and adapt the displacement load mode.
2. When structure is applied fatigue load, it is assumed that *f _n_*(*K* or *G*) is constant during the increase in fatigue crack length (∆*a*)*_n_*. The general Paris law is converted into its incremental form, as shown in Equation (22):
  (22) $${\left(\Delta N\right)}_{n}=\frac{{\left(\Delta a\right)}_{n}}{Cf_{n}{\left(K\;or\;G\right)}^{m}}$$
3. The SERR (*G*) of *n* step can be obtained using FEM methods such as the VCCT and the J-integral method. The procedure of calculation has been introduced in Section 3.1 (once the fatigue load is determined, the SERR (*G*) can be determined). After selecting the function of *f* (*K* or *G*), *f_n_*(*K* or *G*) around the crack tip in *n* step can be calculated.
4. Up till now, (∆*a*)*_n_* and *f_n_*(*K* or *G*) can be obtained from the previous steps. After substituting (∆*a*)*_n_*, *f_n_*(*K* or *G*), and known experimental fitting coefficients *C* and *m* into Equation (22), (∆*N)_n_* can be finally obtained. It should be noticed that *C* and *m* can present material constants or functions of parameters, such as fatigue crack length (*a*).
5. Updating fatigue cycle number *N_n+1_* of the (*n* + 1) step using Equation (23). The structural stiffness declines as a result of delamination damage. Go back to step 2 and calculate delamination propagation of next incremental step until it stops when *f_n_*(*K* or *G*) ≤ *f_n_*(*K* or *G*)_th_. Record the final fatigue crack length (*a*) and fatigue cycle (*N*). *f_n_*(*K* or *G*)_th_ is the inherent properties of the material, which is obtained by experimenting with the fatigue crack growth rate.
  (23) $$N_{n+1}=N_{n}+{\left(\Delta N\right)}_{n}$$

Paris law is directly applied to fatigue delamination in finite element software. The life prediction of composite laminated structures can be completed as long as the general formulas summarized in Section 3.3 are used to construct the modified Paris law. In addition, various influence parameters mentioned before can be considered. Moreover, the data of material systems should be fitted through experiments. 

However, the fracture mechanics model also has its limitations [124]: (1) The essence of the FEM is to discretize structure, and the increment of fatigue crack length (∆*a*) is usually equal to the characteristic length of the element (*L*_c_). The higher the mesh density, the higher the accuracy of the fatigue crack growth curve (*da*/*dN*-∆*G*). Therefore, this model is greatly sensitive to mesh size. (2) Coefficients of the Paris law are parameters of material properties. Thus, it is difficult to accurately simulate fatigue delamination at the interface of mixed materials. (3) The FEM needs the growth paths of pre-cracks in advance. Moreover, it is not able to predict delamination onset or simulate spatial cracking, as there are no parameters under the traditional Paris law to determine the direction of delamination propagation.

### 4.3. Damage Mechanics Model

**Figure 14:**
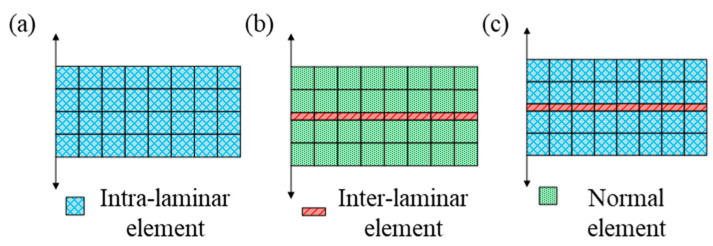
Fatigue delamination model based on damage mechanics. (**a**) Fatigue delamination model based on the degradation of intra-laminar elements. (**b**) Fatigue delamination model based on the degradation of inter-laminar elements. (**c**) Fatigue delamination model based on the degradation of hybrid intra-laminar elements and inter-laminar elements.

The damage mechanics model can directly predict delamination onset and propagation [113]. Thus, compared with the fracture mechanics model, it has been paid more attention in recent years. At present, numerical methods of fatigue delamination based on damage mechanics can be classified into three types: (1) FDDMs that only capture the intra-laminar damage mechanisms, which ignore inter-laminar delamination; (2) FDDMs that only support the degradation of inter-laminar elements, which ignore intra-laminar damage; and (3) FDDMs that support the degradation of intra-laminar elements and inter-laminar elements simultaneously. It should be noted that the latter two models encompass physical modeling of cohesive elements. Taking mode I delamination as an example, the schematic diagrams are shown in Figure 14.

#### 4.3.1. Intra-Laminar Element Model

**Figure 15:**
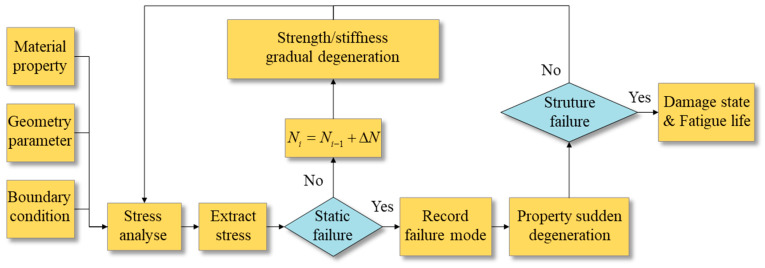
Flow chart of fatigue delamination for the degradation of intra-laminar elements.

Based on the degradation of intra-laminar elements in FDDMs, stresses of intra-laminar elements around the crack tip are substituted into strength criteria to estimate delamination onset. Furthermore, delamination propagation is simulated via damage evolution. The concept and procedure of residual stiffness/strength models and FPDMs are similar. Therefore, the specific analytical process of the residual stiffness/strength model is depicted in Figure 15. 

As the number of fatigue cycles increases, properties of the intra-laminar elements gradually degrade [114]. Stiffness degradation leads to the redistribution of stress fields. Then, the composite laminated structure reaches a higher stress state. Strength degradation alters the parameters of strength in failure criteria. Therefore, local static strength failure occurs first in the structure, and it finally leads to overall structural failure.

In addition to the strength failure criteria for delamination, many investigators have studied the degradation model of material parameters, damage variables [125,126,127], and the damage stiffness matrix [128] on intra-laminar elements. Although the intra-laminar element model can implement the simulation of delamination onset and propagation, the failure mechanism is essentially distinct from the interface of actual structures. Moreover, the stiffness/strength degradation model depends on a large amount of experimental data, which elevates costs. Additionally, the empirical assumptions of damage variables and constitutive models reduce the prediction accuracy of fatigue delamination.

#### 4.3.2. Inter-Laminar Element Model

The cohesive zone model (CZM) is the main method for FDDMs based on the degradation of inter-laminar elements. The history of the CZM can be traced back to the D–B model [129,130], which was established by Dugdale and Barenblatt. The CZM is essentially a phenomenological model. The traction–separation law of cohesive elements is a phenomenological description of the fracture process around the crack tip. The CZM has the simplest expression of constitutive equation and clear physical meaning. What is more, it is easy to be realized in ABAQUS software 6.14. Therefore, fatigue delamination simulations based on cohesive elements have been rapidly developed. The basic procedures of the inter-laminar element model under fatigue load are as follows:

(1) Derive the stiffness matrix and the constitutive equation (that is the cohesive law) of cohesive elements.

**Figure 16:**
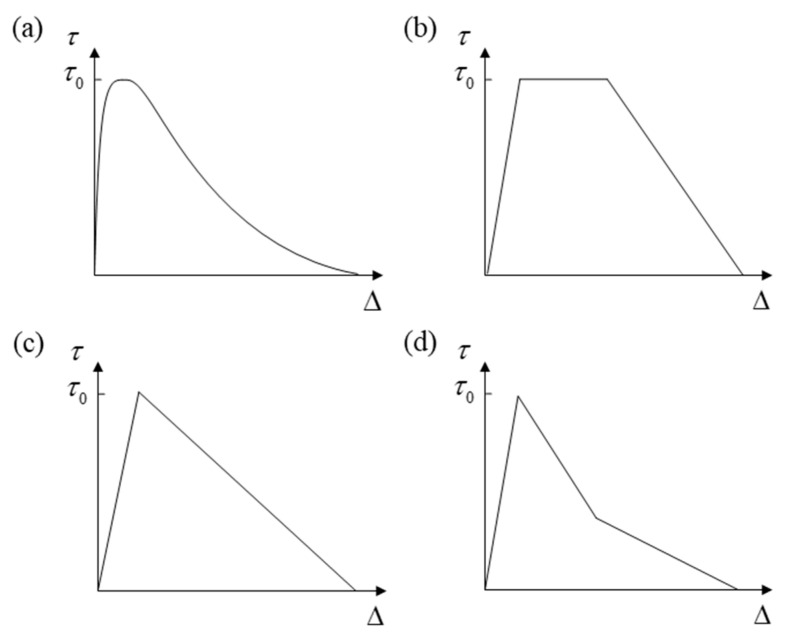
Different constitutive relations. (**a**) Exponent law. (**b**) Trapezoidal law. (**c**) Bi-linear law. (**d**) Tri-linear law.

Davila and Camanho et al. proposed an interface element between solid elements, which helps researchers in understanding how delamination develops and how it can affect the residual performance of delamination [131]. This element has been incorporated into ABAQUS software 6.14. The cohesive element established by Jiang et al. [132] considered the change in the mixed-mode ratio during delamination propagation. What is more, the numerical results were found to be in good agreement with the experimental results. In terms of the cohesive law, Needleman [133] proposed the exponential law; Tvergaard and Hutchinson [134] proposed the trapezoidal law; Mi et al. [135] proposed the bilinear law; and Heidari-Rarani et al. [24] proposed the trilinear law. These constitutive relations are shown in Figure 16. However, relevant studies [136] have indicated that different forms of these constitutive relations have no obvious effect on the load–displacement curves of composite laminated structures. To sum up, the bilinear law has a good balance of computational efficiency, accuracy, and convergence. Therefore, most of the FDDMs based on cohesive elements have generally employed the simplest bilinear softening constitutive relations.

(2) Determine the damage initiation and evolution criteria of mixed-mode fatigue delamination.

At present, the damage initiation criteria of delamination, which are integrated in ABAQUS, include the maximum principal stress/strain criterion, the maximum nominal stress/strain criterion, and the quadratic nominal stress/strain criterion. The damage evolution criteria of delamination include the power law, B–K law [137], and Reeder law [138], in which Reeder law can automatically degenerate to power law when *G*_IIC_ = *G*_IIIC_ [6]. Turon et al. [123] proposed a delamination initiation criterion that considers the effect of crack closure and satisfies the condition of thermodynamic consistency. By controlling the relationship between interface strength and stiffness, a method for accurately predicting mixed-mode delamination propagation has been developed [117]. The common criteria introduced above have been summarized in Table 2. 

**Figure 17:**
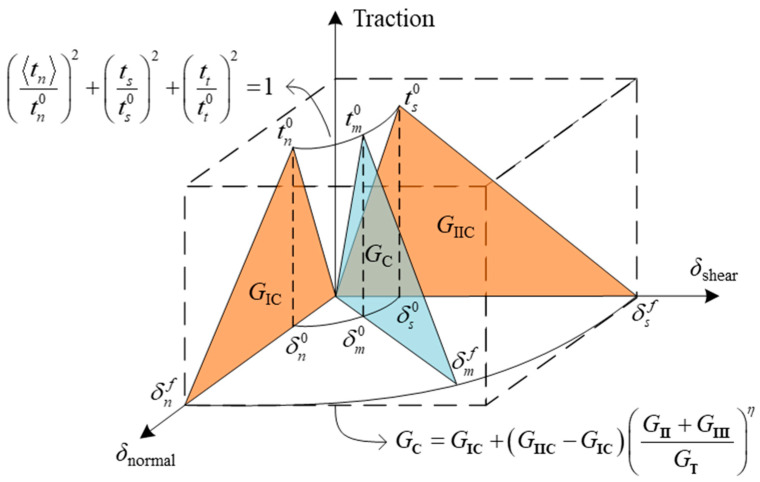
The quadratic nominal stress criterion and B–K law.

Figure 17 depicts the quadratic nominal stress criterion and B–K law of mixed-mode delamination, which are most commonly used. The black solid line derived from the arrow in Figure 17 is the envelope of damage initiation and evolution of cohesive elements. Therefore, different damage initiation and evolution criteria of delamination should be constructed according to material systems; it is a guarantee of accurately monitoring the damage initiation and critical fracture toughness under mixed-mode delamination.

(3) Choose the strategy of FDDMs based on loading–unloading hysteresis [11,41,139] or envelope load (cycle jump approach) [140,141].

**Figure 18:**
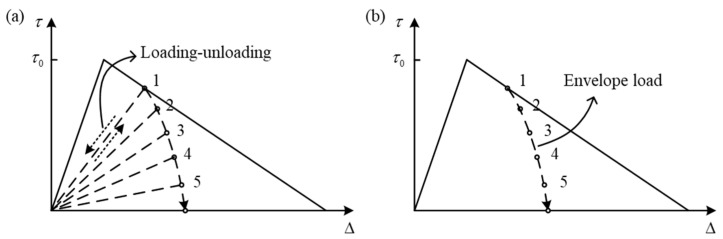
Loading–unloading simulation model [143]. (**a**) Loading–unloading hysteresis damage model. (**b**) Envelope load damage model.

The patterns of inter-laminar property degradation under fatigue load are shown in Figure 18; it illustrates the difference between two kinds of models. It is necessary to calculate the degraded parameters of each cycle in the loading–unloading hysteresis damage model. This method is not desirable under high-cycle fatigue delamination due to its extremely high computational cost. However, only the maximum value of the load block is considered in the envelope load damage model. It has been assumed that the damage is continuous and average in each load block [142], which greatly improves the efficiency of simulations. 

At present, most damage models that are based on the CZM adopt the method of envelope load. Zhang et al. [143] predicted the fatigue delamination of composite laminates using twin cohesive models and a combined static and fatigue cohesive formulation. Compared with the traditional envelope load method, the twin cohesive model is subjected to peak and valley envelope loads, which means that the effect of the load ratio is considered in this model. At the same time, it is significant that this novel model improves the computational efficiency of fatigue delamination.

(4) Establish the differential relationship between the damage variables and the number of fatigue cycles. Then, a reasonable formula for the fatigue delamination damage rate can be obtained.

In the situation of applying the envelope load method to simulate fatigue delamination, the total damage rate of delamination (*dD*/*dN*) is the sum of the quasi-static damage rate (*dD_s_*/*dN*) and the fatigue damage rate (*dD_f/_dN*). Assuming that the current number of fatigue cycles is *N*, the damage variable is updated by Equation (24) after the increment of fatigue cycles (∆*N*). Generally, the left rectangle formula [20,55,144] and trapezoid formula [145] are used to numerically integrate Equation (24) to obtain the damage variable under current fatigue cycles:

(24) $$D(N+\Delta N)=D(N)+\int_{N}^{N+\Delta N}\frac{dD}{dN}dN$$

Robinson et al. [146] proposed a fatigue damage rate model with three parameters. The curves of the fatigue crack growth rate obtained via simulation were consistent with the fitted Paris curves obtained from experimental data. Tumino and Cappello [145] developed a fatigue damage rate model with two parameters, which also reproduced the phenomenon of fatigue delamination tests. The difference is that one of the parameters is a function of the mixed-mode ratio. In the three-parameter model established by Khoramishad et al. [55], both the crack growth threshold and the mixed-mode ratio were simultaneously taken into account. Thus, it is more applicable for engineering than the former models. It should be noted that the parameters of FDDMs are purely based on damage mechanics and cannot be directly obtained via the standard tests. Therefore, the fitting parameters of the model can only be adjusted using the FEM calibration method [147,148,149] to reproduce the crack growth curve of fatigue delamination. At present, the correlation between this model and its structural geometry has not been clearly clarified. Hence, it hinders the application of the model to engineering for predicting fatigue delamination life.

(5) Select the inter-laminar parameters of fatigue delamination damage that need to be degraded. Then, establish the functional relationship between the degradation parameters and the damage variables.

**Figure 19:**
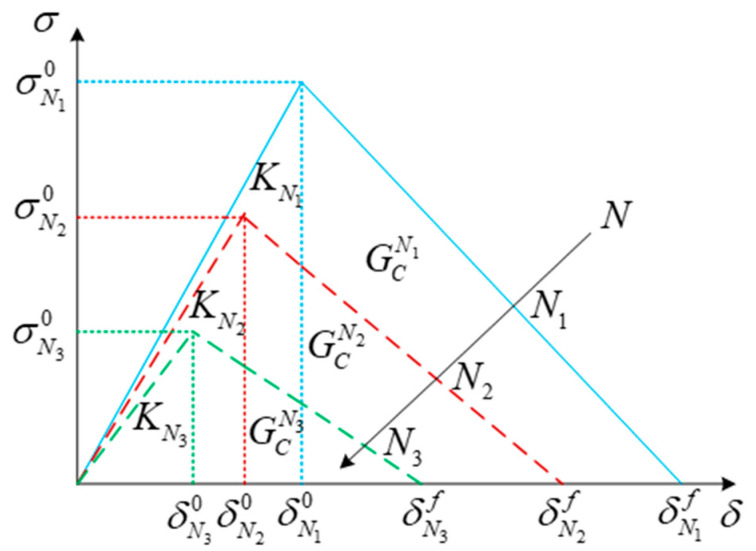
Bilinear softening fatigue delamination model [149].

In the CZM, there are only three inter-laminar parameters. Penalty stiffness (*K*), interfacial strength (σ°), and critical fracture toughness (*G*_c_) are required to describe a bilinear cohesive relationship. The degradation of inter-laminar cohesive elements is carried out to establish a functional relationship between *K*, σ°, and *G*_c_ and a damage variable (*D*(*N*)). Furthermore, this function is applied for softening cohesive law. The simplest softening curve of the bilinear constitutive model [149] is shown in Figure 19.

The process of applying the bilinear softening law is simple. It inherits the concept of residual strength/stiffness of metal fatigue and defines the variables of residual penalty stiffness, interface, strength, and fracture toughness, which are named after $K_{N_{i}}$,$\sigma_{N_{i}}^{0}$, and $G_{C}^{N_{i}}$ in Figure 19. This model assumes that the initial bilinear constitutive relationship of cohesive elements is always maintained during fatigue cycles. With the number of fatigue cycles increasing, its corresponding parameters, such as penalty stiffness, interfacial strength, and fracture toughness, continuously degrade. In other words, these residual parameters, which have been defined based on FDDMs, are related to the fatigue cycles (*N*). Therefore, as long as the relationship between the damage parameters of inter-laminar elements and the fatigue cycles is given, a complete FDDM can be established.

The residual and initial inter-laminar parameters have been set as *R*(*N*) and *R*(*N*)^0^, respectively. What is more, the most commonly used degradation function of inter-laminar performance is as follows:

(25) $$R(N)=(1-D)R{\left(N\right)}^{0}$$

The model established by Turon et al. [117] employed interface strength as a degradation parameter, while the model proposed by Chen et al. [150] applied penalty stiffness as a degradation parameter. The model established by Mazaheri and Hosseini et al. [17] simultaneously degraded six inter-laminar properties corresponding to mode I and mode II delamination. The performances of these models were consistent with those of Roe and Siegmund [151]. Since the function of degradation is empirical, there is no specific correlation between the degradation of inter-laminar parameters and the physical mechanism of fatigue delamination.

(6) Realize the loading/unloading cycles of FDDMs using ABAQUS software 6.14 and formulate the standard of integral structure failure. Finally, fatigue cycles of delamination under a specific fatigue load can be calculated.

**Figure 20:**
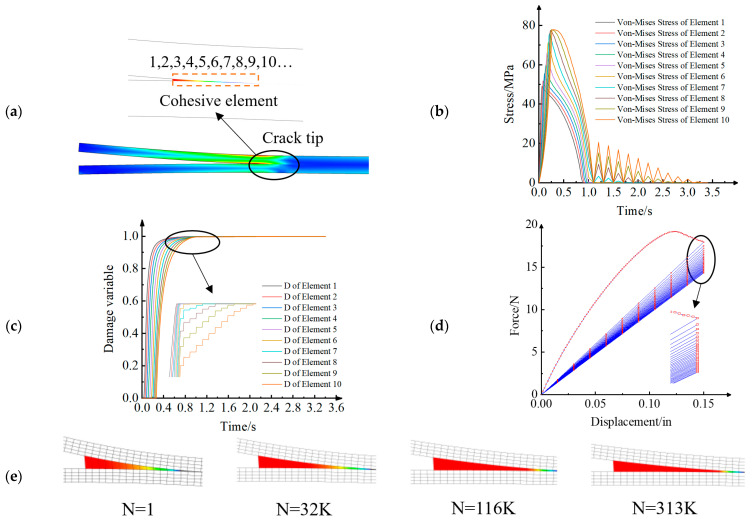
FEM simulation results of DCB fatigue delamination damage. (**a**) DCB model. (**b**) Mises stress of elements from the crack front. (**c**) Damage variables of elements from the crack front. (**d**) Load–displacement curve of DCB fatigue delamination. (**e**) Delaminated fatigue crack propagation and lifespan.

Taking the fatigue delamination of a DCB as an example, a modified FDDM can be established by reproducing the model proposed by Koloor et al. [149]. The DCB in this example had a span of 9.0 in (228.6 mm) with a rectangular cross-section of 1.0 in (25.4 mm) wide × 0.4 in (10.2 mm) deep. One end of the beam was fixed, and the displacements were applied at the other end. This model has matched meshes with 90 × 4 meshes for each half of the DCB. More details on the geometry and materials of the specimen are in the ABAQUS 6.14 documentation [152] and handbook [153]. As shown in Figure 20, the results of the FEM were obtained via the UMAT subroutine in ABAQUS. The model was loaded with a prefabricated crack within the first second. Afterwards, the zigzag wave with a frequency of 5 Hz was applied. The load mode was the displacement control. It should be noted that the number of simulated cycles does not correspond to the actual number of fatigue cycles. General fatigue programs require the use of acceleration algorithms [20,140,141]. Figure 20a shows the local stiffness degradation at the fatigue crack front of the DCB specimen. Figure 20b presents the von Mises stress of 10 cohesive elements around the crack front. The smaller the element number, the closer it is to the crack front. It is obvious that the first five elements have already failed during the pre-cracking stage. The last five elements gradually failed under fatigue delamination. Corresponding to Figure 20b, Figure 20c clearly demonstrates the evolution process of damage variables, which represents the damage degree of the element around the crack tip. Due to the displacement control, as shown in Figure 20d, the descending speed of the load–displacement curve gradually slows down and finally tends to stop. This is consistent with the delamination crack growth rate that is shown in Figure 20e.

**Figure 21:**
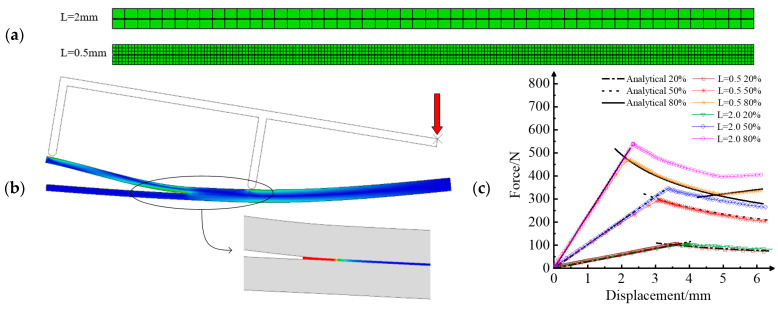
Effect of the mesh size on the load–displacement curve of the MMB test. (**a**) MMB with different mesh sizes; (**b**) MMB model; and (**c**) comparison of results for L = 0.5 mm and L = 2.0 mm.

In order to realize the implementation of fatigue delamination crack growth with the cohesive element model, the sensitivity analysis of parameters has been carried out on the basis of quasi-static delamination damage. Gustafson and Waas et al. [154] studied the influences of penalty stiffness, inter-laminar strength, fracture toughness, and constitutive shape on the FEM results for the DCB and ENF. The results indicated that the DCB model mainly depends on one parameter, while the ENF model depends on multiple parameters. The same was also true: the constitutive shape has little influence on the results of the model. Turon et al. [155] found that inappropriate parameters may lead to the formation of large errors between simulations and experiments. Therefore, a penalty stiffness model with variation of the mixed-mode ratio was developed to adjust the model parameters, which dynamically matched the results of the experiment. In order to better illustrate the influence of parameters on numerical examples, the constitutive relationship of cohesive elements was realized in the UMAT subroutine. The accuracy of the program was verified by comparing it with the models in the ABAQUS documentation. Figure 21 depicts the sensitivity of the mesh size in numerical simulations of MMB. The geometry of the specimens and the material properties are presented in references [131,156]. In Figure 21a, different mesh sizes (L = 2 mm and L = 0.5 mm) of MMB are presented. In Figure 21b, the typical stress nephogram of the MMB model and the damage variable nephogram of local cohesive elements are given, and the red arrow represents the loads applied. Figure 21c visually presents the influence of the mesh size on the load–displacement curve of MMB under different mixed-mode ratios. It can be seen that the influence of mesh size is non-negligible. A large mesh size (L = 2 mm) increases the structural stiffness and overestimates the ultimate load of MMB. Therefore, the convergence of the critical load and displacement is sensitive to the element size. 

#### 4.3.3. Mixed Intra-Laminar/Inter-Laminar Element Model

Numerical simulations of fatigue delamination based on the mixed intra-laminar/inter-laminar element model are no longer repeated in detail. The degradation of intra-laminar and inter-laminar elements are considered simultaneously in the FEM. In addition, the fatigue damage of two different kinds of element affects each other.

Du et al. [12] combined the intra-laminar elements of Linde et al. [128] with the exponential cohesive law by referring to an example of tensile failure of fiber–metal laminates (FMLs) in the ABAQUS documentation. The delamination failure analysis of Ti/Cf/PEEK laminates was carried out. Tarfaoui et al. [157] studied the progressive delamination damage failure of CFRP composites with open holes via different failure criteria for intra-laminar elements and cohesive elements. Aoki et al. [34] classified FPDMs of composite laminated structures into two types: the intra-laminar damage model and the inter-laminar damage model. The fatigue tests of the DCB, ENF, and curved laminates verified the accuracy of the model with respect to the life prediction of CFRP composite laminates under different fatigue load levels.

The mixed intra-laminar/inter-laminar element model takes the fatigue damage of intra-laminar elements into account while simulating the damage evolution of inter-laminar elements. In particular, with regard to composite laminated structures, which have complex stacking sequences and geometries, coupling effects always accelerate the speed of fatigue delamination crack growth and make fatigue life predictions closer to the actual conditions. However, this model also has similar issues to the inter-laminar element model and intra-laminar element model. It is inevitable that this model has limitations in obtaining parameters based on numerical simulation and empirical degradation assumptions, which restricts the development of the model to some extent.

### 4.4. Hybrid Model

In the study of FDDMs, models that are based on damage mechanics are not able to essentially reflect the degradation properties of laminated composite structures under fatigue load. What is more, it does not establish the relationship between the fatigue delamination growth rate in numerical simulations and standard experiments. Investigators have started to combine CZMs based on damage mechanics with Paris law models based on fracture mechanics. They have explored a general method of establishing FDDMs based on the fatigue delamination experiments of composite laminated structures.

The procedure based on hybrid fractures and the damage mechanics model can refer to damage mechanics models in Section 4.3. In step 4, the fatigue damage rate (dD/dN) is related to the fatigue crack growth rate (da/dN) of Paris law via the chain rule [35]. The final general formula for the fatigue damage rate is shown in Equation (26) below:

(26) $$\frac{dD}{dN}=\frac{dD}{da}\frac{da}{dN}=\frac{dD}{da}Cf{\left(G\right)}^{m}$$

**Figure 22:**
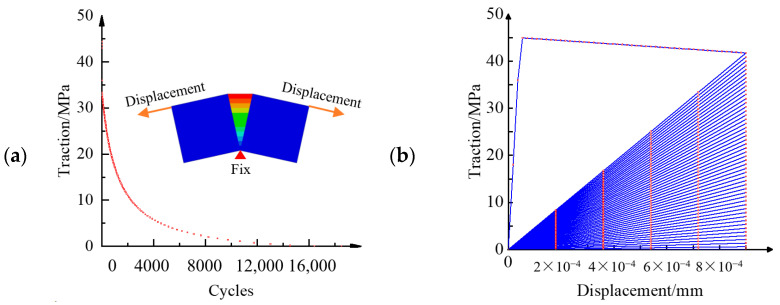
Cohesive element test of high-cycle fatigue model. (**a**) Cohesive element model and traction–cycles curve. (**b**) Traction–displacement curve of an element under fatigue load.

Turon et al. [20] proposed a damage model to simulate delamination propagation under high-cycle fatigue loads. The relationship between the damage variables and the crack growth rate in Paris law was established using cohesive elements. The verification of element was carried out using the UMAT subroutine in ABAQUS. Furthermore, the results of the model was verified by comparing it with fatigue delamination experiments. At the same time, it is also the basic program for many researchers in this field to deeply study the fatigue delamination damage of interface elements. The performances of cohesive elements reproduced using the UMAT subroutine in ABAQUS are given in Figure 22. The finite element model consists of two 4-node plane strain elements connected via a 4-node cohesive element. The load is controlled by displacement. The first load step was a quasi-static step, where the displacement was loaded up to 20 times the delamination onset. The second load step accounts for fatigue damage resulting from a maximum applied displacement of 20 times the delamination onset and a load ratio of *R* = 0. The specific geometry and properties used in the model can be found in reference [20]. It should be noted that a cycle jump strategy [20,158] was implemented, and that the maximum interval of the damage variable (∆d_max_) was set as 0.001 in this model. Figure 22a shows the cohesive element model and the traction–cycles curve. It is obvious that the traction of cohesive elements experiences an initial sudden drop and then a slow drop. Figure 22b shows the traction–displacement curve of a cohesive element. The cohesive element fails after about 13 K cycles of fatigue loading.

Harper and Hallett [159] developed a degradation law of cohesive elements for delamination propagation under fatigue load. The model directly related the SERR of the cohesive zone to the Paris law curve that was obtained from the experiment. Thus, it reproduced the phenomenon of delamination propagation. The results obtained from the proposed fatigue delamination model were in good agreement with the experimental data of the DCB, 4ENF, and MMB. Nixon-Pearson et al. [160] established a cohesive element based on Paris law to simulate the relationship of delamination and matrix cracking for composite laminates with open holes. The S–N curve was consistent with the experimental results. Due to the sensitivity of the hybrid fracture and damage mechanics model to mesh size, Tao et al. [161] proposed a method that applies a virtual fatigue damage variable to locate elements around the crack tip. By combining the modified Paris law and the extended cohesive element, a hybrid model with a weak dependency on mesh size was obtained. The numerical simulation was able to reproduce the results of the DCB and 4ENF under fatigue load. Ebadi-Rajoli et al. [162] developed a new fatigue damage accumulation (FDA) model by correlating cohesive elements based on fracture mechanics and damage mechanics and adopting the degradation of interface stiffness. 

Compared with damage mechanics models, the hybrid fracture and damage mechanics models based on Paris law do not need parameter calibration of numerical simulations to match the fatigue delamination growth rate. On account of the Paris law based on the theoretical framework of LEFM, it is an inherent property of material and does not depend on the geometry of the specimen. Therefore, the fatigue delamination growth rate curve of the standard specimen, which is the input parameter of the hybrid model, reflects the phenomenological model summarized from experiments. In addition, the general modified Paris law, which considers the influence factors of fatigue delamination propagation in Section 3.3, can arbitrarily form a hybrid fracture and damage mechanics model with the CZM. Its high generality and operability can flexibly satisfy requirements of research and engineering.

The fatigue delamination models mentioned above in this section have all been proposed for monitoring the delamination growth of inter-laminar cohesive elements, which neglect the degradation of intra-laminar elements. However, it is difficult for these models to predict the delamination propagation of angle-ply laminates with open holes under fatigue load. This is due to the fact that the coupling effect of matrix cracking and fiber-matrix shear failure in such complex laminated composites is significant. Therefore, it is natural to combine intra-laminar elements based on a strength criterion and inter-laminar cohesive elements based on the modified Paris law. Zhou et al. [14] developed such a hybrid model and carried out the fatigue life prediction of GFRP and CFRP composite joints to validate the accuracy of the model. This kind of model synthesizes strength criteria, fracture mechanics, and damage mechanics. It exhibits strong structural universality and engineering practicability in theory. However, more standard experiments are needed to support the input parameters of this model, which has the limitations of low efficiency and high cost.

## 5. Summary and Prospects

Based on the phenomenon of composite laminated structures under fatigue load, this review discusses the general methods of establishing phenomenological models for fatigue delamination. It focuses on the influence factors and correction techniques of Paris law. What is more, the classification, procedure, performance, and limitations of common FDDMs are also discussed.

As a phenomenological model based on the theoretical framework of LEFM, Paris law has been widely studied for considering the effects on fatigue delamination life. Its influences include the mixed-mode ratio, load ratio, threshold of fatigue delamination growth rate, fiber bridging, temperature, humidity, and load sequence. The cohesive element model based on damage mechanics has clear physical meaning and simple constitutive relation. Therefore, the FDDMs based on the modified Paris law within the theoretical framework of hybrid fracture and damage mechanics are the most effective and stable method, so far, to simulate fatigue delamination onset and propagation.

However, the phenomenological model is not enough to clearly explain the fatigue delamination mechanism of composite materials. The establishment of FDDMs mostly depends on the accumulation of experimental data and the assumption of semi-empirical formulas. Thus, it is difficult to obtain accurate results when studying complex structures and load conditions, which greatly limits its application in practical engineering. 

Based on the investigation of existing FDDMs, the following directions are suggested according to the aforementioned technical problems to be solved:

(1) The traditional continuum damage models (CDMs) [126] have been extensively used for capturing intra-laminar damage mechanisms like fiber breaking and matrix cracking, which result in the development of CZMs [117] for inter-laminar delamination. However, the simulation accuracy of fatigue delamination in many models greatly depends on the mesh density around the crack tip. Therefore, the multi-scale CZM [163,164] fatigue delamination model for crack tip tracking [21,144] came into existence, which simultaneously considers both global and local models;
(2) As an extension of the traditional FEM, the extended finite element method (XFEM) can expand the degree of freedom (DOF) of elements with displacement functions to obtain discontinuities responses [6]. The XFEM has the ability to predict local stress fields and stress concentration fields accurately without refining meshes. In addition, this method can predict delamination propagation without pre-cracks, which are obvious prior to the CZM [165]. Some investigators have applied the XFEM to analyze fatigue delamination onset and propagation [166];
(3) It is difficult for traditional FDDMs to solve the problem of spatial delamination for angle-ply laminated composite structures. In the last decade, the phase field (PF) approach to brittle fracture has emerged as a reliable alternative method for modeling the progressive failure of composite materials [167,168]. On the basis of the PF, researchers have established a PF-CZM approach [169,170] for modeling the delamination propagation of composite materials;
(4) The vital idea of isogeometric analysis (IGA) is to apply non-uniform rational B-splines (NURBSs) to approximate solution fields in the FEM. Some investigators [171] have found that shear stresses provided by the FEM models exhibit prominent oscillatory behaviors, while the IGA models show fewer oscillations and yield more precise solutions. This implies that there is the potential to employ IGA to track the crack growth of irregular geometric models;
(5) The capture of delamination propagation greatly depends on the accurate prediction of inter-laminar stresses. To the best of the authors’ knowledge, it is extremely effective to obtain inter-laminar stress via the high-order deformation theory (HODT). Some researchers have constructed higher-order composite beam/plate/shell elements [172,173] to predict the delamination and critical load of composite laminated structures;
(6) It can be found that the phenomenological model based on tremendous observations is still an important way to study fatigue delamination onset and propagation. The machine learning models are suitable for cases where the amount of available data is large and the physics-based mathematical approaches are complex. Therefore, it is natural to integrate the machine learning models into the data processing of the phenomenological delamination model [174,175,176,177,178].

## Figures and Tables

**Table 1:** The commonly used microscopic strength failure criteria.

Criterion	Expression of *f*
Hashin	$\begin{array}{l} {\left(\frac{\sigma_{3}}{Z_{T}}\right)}^{2}+{\left(\frac{\sigma_{23}}{S_{23}}\right)}^{2}+{\left(\frac{\sigma_{31}}{S_{31}}\right)}^{2}\geq 1,\;\;\;\;\sigma_{3}>0\\ {\left(\frac{\sigma_{3}}{Z_{c}}\right)}^{2}+{\left(\frac{\sigma_{23}}{S_{23}}\right)}^{2}+{\left(\frac{\sigma_{31}}{S_{31}}\right)}^{2}\geq 1,\;\;\;\;\sigma_{3}<0 \end{array}$
Chang-Chang	$\begin{array}{l} {\left(\frac{\sigma_{33}}{Z_{T}}\right)}^{2}+\frac{\sigma_{31}^{2}/2G_{31}+3/4\alpha \sigma_{31}^{4}}{S_{31}^{2}/2G_{31}+3/4\alpha S_{31}^{4}}+{\left(\frac{\sigma_{23}}{S_{23}}\right)}^{2}\geq 1,\;\;\;\;\sigma_{3}>0\\ {\left(\frac{\sigma_{33}}{Z_{C}}\right)}^{2}+\frac{\sigma_{31}^{2}/2G_{31}+3/4\alpha \sigma_{31}^{4}}{S_{31}^{2}/2G_{31}+3/4\alpha S_{31}^{4}}+{\left(\frac{\sigma_{23}}{S_{23}}\right)}^{2}\geq 1,\;\;\;\;\sigma_{3}\leq 0 \end{array}$
Hou	$\begin{array}{c} {\left(\frac{\sigma_{33}}{Z_{T}}\right)}^{2}+\frac{\sigma_{23}^{2}+\sigma_{13}^{2}}{S_{13}^{2}\left(d_{ms}d_{fs}+\delta \right)}\geq 1,-\sqrt{\left(\sigma_{13}^{2}+\sigma_{23}^{2}\right)/8}\leq \sigma_{33}<0\\ \frac{\sigma_{23}^{2}+\sigma_{13}^{2}-8\sigma_{33}^{2}}{S_{13}^{2}\left(d_{ms}d_{f_{5}}+\delta \right)}\geq 1,-\sqrt{\left(\sigma_{13}^{2}+\sigma_{23}^{2}\right)/8}<\sigma_{33}\\ 0,\;\mathrm{else}\; \end{array}$
Zou	$\frac{Z_{T}}{Z_{C}}{\left(\frac{\sigma_{3}}{Z_{T}}\right)}^{2}+\left(1-\frac{Z_{T}}{Z_{C}}\right)\frac{\sigma_{3}}{Z_{T}}+\frac{\tau_{13}^{2}}{S_{13}^{2}}+\frac{\tau_{23}^{2}}{S_{23}^{2}}\geq 1$
LaRC	$\begin{array}{c} (1-g)\frac{\sigma_{33}}{S_{33}}+g{\left(\frac{\sigma_{33}}{S_{33}}\right)}^{2}+\frac{\chi \left(\tau_{13}\right)}{\chi \left(S_{13}\right)}+\frac{\chi \left(\tau_{23}\right)}{\chi \left(S_{23}\right)}\geq 1,\;\;\;\;\sigma_{22}>0\\ \frac{\chi \left(\tau_{13}\right)}{\chi \left(S_{13}\right)}+\frac{\chi \left(\tau_{23}\right)}{\chi \left(S_{23}\right)}\geq 1,\;\;\;\;\sigma_{22}\leq 0 \end{array}$

**Table 2:** Common damage initiation/evolution criteria of delamination.

Initiation Criterion	f Expression	Evolution Law	f Expression
Maximum principal stress/strain criterion	$\left\{\frac{\sigma_{\max}}{\sigma_{\max}^{0}}\right\},\;\left\{\frac{\varepsilon_{\max}}{\varepsilon_{\max}^{0}}\right\}$	Power law	${\left(\frac{G_{I}}{G_{\mathrm{IC}}}\right)}^{\alpha }+{\left(\frac{G_{\mathrm{II}}}{G_{\mathrm{IIC}}}\right)}^{\beta }+{\left(\frac{G_{\mathrm{III}}}{G_{\mathrm{IIIC}}}\right)}^{\gamma }$
Maximum nominal stress/strain criterion	$\begin{array}{l} \max\left\{\frac{\langle t_{n}\rangle }{t_{n}^{0}},\frac{t_{s}}{t_{s}^{0}},\frac{t_{t}}{t_{t}^{0}}\right\}\\ \max\left\{\frac{\varepsilon_{n}}{\varepsilon_{n}^{0}},\frac{\varepsilon_{s}}{\varepsilon_{s}^{0}},\frac{\varepsilon_{t}}{\varepsilon_{t}^{0}}\right\} \end{array}$	B–K law	$\frac{G_{T}}{G_{\mathrm{IC}}+\left(G_{\mathrm{IIC}}-G_{\mathrm{IC}}\right){\left(\frac{G_{\mathrm{II}}+G_{\mathrm{III}}}{G_{T}}\right)}^{\eta }}$
Quadratic nominal stress/strain criterion	$\begin{array}{l} {\left(\frac{\langle t_{n}\rangle }{t_{n}^{0}}\right)}^{2}+{\left(\frac{t_{s}}{t_{s}^{0}}\right)}^{2}+{\left(\frac{t_{t}}{t_{t}^{0}}\right)}^{2}\\ {\left(\frac{\varepsilon_{n}}{\varepsilon_{n}^{0}}\right)}^{2}+{\left(\frac{\varepsilon_{s}}{\varepsilon_{s}^{0}}\right)}^{2}+{\left(\frac{\varepsilon_{t}}{\varepsilon_{t}^{0}}\right)}^{2} \end{array}$	Reeder law	$\frac{G_{T}}{G_{\mathrm{IC}}+\left[\left(G_{\mathrm{IIC}}-G_{\mathrm{IC}}\right)+\left(G_{\mathrm{IIIC}}-G_{\mathrm{IIC}}\right)\left(\frac{G_{\mathrm{III}}}{G_{\mathrm{II}}+G_{\mathrm{III}}}\right)\right]{\left(\frac{G_{\mathrm{II}}+G_{\mathrm{III}}}{G_{T}}\right)}^{\eta }}$

## Data Availability

Not applicable.
